# Genomic footprints related with adaptation and fumonisins production in *Fusarium proliferatum*

**DOI:** 10.3389/fmicb.2022.1004454

**Published:** 2022-09-21

**Authors:** Ling Wang, Qing Liu, Shuailing Ge, Wenhao Liang, Weiyang Liao, Wen Li, Guiai Jiao, Xiangjin Wei, Gaoneng Shao, Lihong Xie, Zhonghua Sheng, Shikai Hu, Shaoqing Tang, Peisong Hu

**Affiliations:** State Key Laboratory of Rice Biology, China National Center for Rice Improvement, China National Rice Research Institute, Hangzhou, China

**Keywords:** *Fusarium proliferatum*, rice spikelet rot disease, fumonisins, genome resequencing, genetic variation, genome-wide association study

## Abstract

*Fusarium proliferatum* is the principal etiological agent of rice spikelet rot disease (RSRD) in China, causing yield losses and fumonisins contamination in rice. The intraspecific variability and evolution pattern of the pathogen is poorly understood. Here, we performed whole-genome resequencing of 67 *F. proliferatum* strains collected from major rice-growing regions in China. Population structure indicated that eastern population of *F. proliferatum* located in Yangtze River with the high genetic diversity and recombinant mode that was predicted as the putative center of origin. Southern population and northeast population were likely been introduced into local populations through gene flow, and genetic differentiation between them might be shaped by rice-driven domestication. A total of 121 distinct genomic loci implicated 85 candidate genes were suggestively associated with variation of fumonisin B1 (FB1) production by genome-wide association study (GWAS). We subsequently tested the function of five candidate genes (*gabap*, *chsD*, *palA*, *hxk1*, and *isw2*) mapped in our association study by FB1 quantification of deletion strains, and mutants showed the impact on FB1 production as compared to the wide-type strain. Together, this is the first study to provide insights into the evolution and adaptation in natural populations of *F. proliferatum* on rice, as well as the complex genetic architecture for fumonisins biosynthesis.

## Introduction

Rice spikelet rot disease (RSRD), also called pecky rice or kernel spotting, is one of the fastest spreading diseases in China, particularly along the middle and lower reaches of the Yangtze River (Huang et al., 2011a). It was categorized as a minor disease that occurred only sporadically before 2006. After that, RSRD epidemics became more frequent and serious due to the extensive cultivation of *japonica* varieties and *indica*-*japonica* hybrids (Wang L. et al., 2022). *Fusarium proliferatum* (teleomorph: *Gibberella fujikuroi*) is the most predominant pathogenic fungus associated with RSRD (Huang et al., 2011b; Wang et al., 2021). Rice is highly susceptible to *F. proliferatum* during the flowering stage (Sun et al., 2019a). After spore germination, the fungal hyphae enter glumes through the anthers at the pollen-filling stage, proceed to colonize pistils and endosperms, and finally infect the whole floral tissues (Sun et al., 2019b). The initial lesions of spikelet rot symptom are reddish-brown or rust-red on rice spikelets at the flowering and milking stage, eventually become blackish-brown during the ripening stage. High precipitation, relative humidity, and warm temperature during anthesis in rice favor the development of RSRD. The percentage of diseased panicles can reach up to 80% under the severe situation of the epidemic (Li et al., 2015). To date, RSRD has been estimated to occur in one third of rice-growing regions in China, resulting in yield losses of 5–30% (Wang L. et al., 2022). How and why the disease emerged in rice is still a matter of speculation. Examination of the spatial and temporal dynamics of disease progression that is prerequisite to develop risk assessments and provide the effective management approaches.

*F. proliferatum* is well known for its pathogenicity to a wide range of cereals, such as wheat, rice, barley, maize, rye and oats. Moreover, the pathogen is notorious for producing multiple mycotoxins, including fumonisins, moniliformin, fusaric acid, fusarin C, and beauvericin (Studt et al., 2012). Fumonisins are associated with multiple human and animal diseases, as they are potent inhibitors of sphingolipid metabolism in eukaryotes (Braun and Wink, 2018). Fumonisins induce equine leukoencephalomalacia in horses, pulmonary edema in pigs, as well as liver and kidney toxicities in rats (Riley and Merrill, 2019). Epidemiological evidences indicated a causal relationship between high levels of fumonisins exposure and neural tube defects and esophageal cancer in human beings (Yu et al., 2021). A widespread survey of fumonisins contamination on a global scale in the post-harvest grain foods ranged from 39% in Europe to 95% in North and South America (Lee and Ryu, 2017). Fumonisins comprise a group of 28 analog, which can be separated into four main groups, fumonisin A, B, C, and P (Braun and Wink, 2018). Fumonisin B (FB) analogs include FB1, FB2, and FB3, with FB1 being the most prevalent component and usually being found at the highest levels (Kamle et al., 2019). International Agency for Research on Cancer (IARC) classified FB1 as a class 2B possible carcinogen for human (International Agency for Research on Cancer [IARC], 1993). The European Union had set maximum limits for fumonisins in food and feedstuffs intended for human consumption (European Commission [EC], 2006). In order to prevent the reproduction and contamination of *F. proliferatum* as efficiently as possible, the control methods should be implemented in dependence on the fungi growth, reproduction, infection and mycotoxins formation in the agricultural fields.

Currently, it is known that the activation of biosynthesis pathways of secondary metabolites (SMs) not only depends on pathway-specific regulators, but also on global transcriptional complexes, signal transduction regulation, and transcriptional and epigenetic manipulation (Brakhage, 2013; Keller, 2019). Fumonisins are a family of polyketide synthases (PKS) derived SMs, consisting of a linear 20-carbon aminopolyhydroxyalkyl chain backbone. The 42-kb long cluster of fumonisin biosynthetic genes (*FUM*) had been well characterized in *Fusarium verticillioides* (Brown et al., 2007; Proctor et al., 2013). *FUM* genes are usually silent or expressed at very low levels under non-inducing conditions. Several external triggers, such as nutritional input (Kim and Woloshuk, 2008), light regulation (Wiemann et al., 2010), ambient pH (Flaherty et al., 2003), and water activity (Cendoya et al., 2018), had been characterized to be involved in fumonisins production. It is therefore necessary to decipher the key molecular players regulating the biosynthesis of fumonisins, as well as the interplay between the lifestyle of toxigenic fungi and genetic constitution.

To date, no reports have been elucidated about the biology and genetics of *F. proliferatum* population from rice. In this study, we reported the genome sequences of 67 *F. proliferatum* strains collected from major rice-growing regions in China, in order to characterize its genetic diversity and population structure, and dissect the genetic determinants involved in fumonisins biosynthesis. The findings of this study provided a genome-wide perspective on the population of *F. proliferatum* and clarified their genetic relationships with fumonisins production.

## Materials and methods

### Fungal strains and growth conditions

**FIGURE 1:**
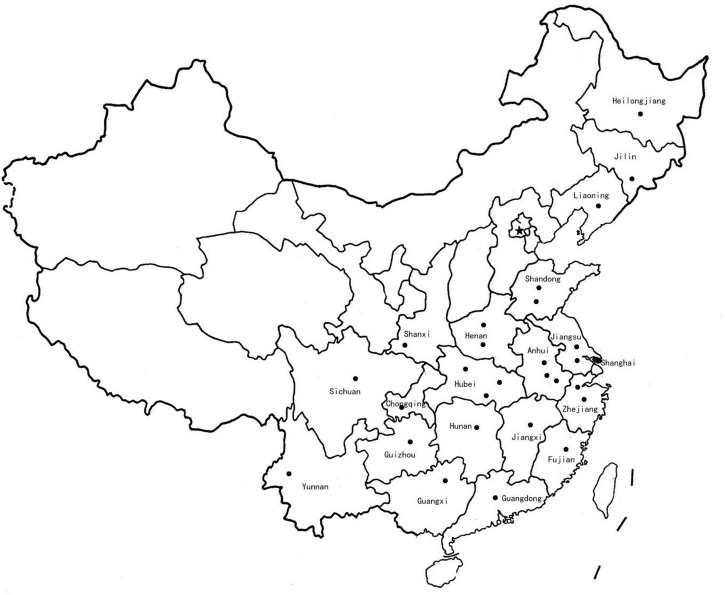
Map of China showing the location (dots) where *Fusarium proliferatum* strains were collected.

The *F. proliferatum* strains were isolated from naturally infected spikelets of rice at different geographical locations in China (Figure 1 and Table 1). The monoconidial culture was grown on potato dextrose agar (PDA, 20% potato extract, 2% dextrose, 1.5% agar) and minimal media [MM: 1% (w/v) carbon source, 50 ml of a 20 × salt solution (120 g/l NaNO_3_, 10.4 g/l KCl, 30 g/l KH_2_PO_4_, and 10.4 g/l MgSO_4_), and 1 ml of 5 × trace elements (22.0 g/l ZnSO_4_, 11 g/l boric acid, 5 g/l MnCl_2_, 5 g/l FeSO_4_, 1.6 g/l CoCl_2_, 1.6 g/l CuSO_4_, 1.1 g/l (NH_4_)_2_MoO_4_, and 50 g/l EDTA), pH 6.5] at 28°C. The test of the evaluation of different carbon sources included the adding to the MM of glucose, sucrose, fructose, maltose and galactose. PDA media supplemented with H_2_O_2_ (3 mM), sodium dodecyl sulfonate (SDS, 0.025%) and calcofluor white (CW, 50 μg/mL) were used for stress response assays. Conidia were obtained in YEPD liquid media (0.5% yeast extract, 1% peptone and 2% glucose) and stored into 20% glycerol at −80°C for long term storage. Species identification was performed based on colony morphology and PCR amplification as previously described (Mulè et al., 2004). Mating-type (MAT) idiomorph was determined by blasting the known MAT gene sequences (Steenkamp et al., 2000). The chitin content was measured by percentage of N-acetylglucosamine (GlcNAc) relative to the dry mycelial weight (Kong et al., 2012).

**TABLE 1:** Fumonisin FB1 production and variant calling statistics of *Fusarium proliferatum.*

Strains	Geographic origin	FB1 content	MAT type	SRA ID	Reads depth (X)	Genome coverage (%)	SNPs	Indels	Exonic variants	Intronic variants	Non-genic variants	Splice site variant	Variant density (/kb)
A1	Hangzhou, Zhejiang	1.7	1–1	SRS4609098	55.7	99.68	80,361	7	46,721	4,084	28,412	1,144	2.07
A2	Hangzhou, Zhejiang	1.7	1–1	SRS4609317	58.3	99.63	80,766	2	46,492	4,145	28,991	1,138	2.08
A3	Hangzhou, Zhejiang	1.4	1–2	SRS4609316	56.4	99.59	68,620	2	39,838	3,515	24,279	988	1.77
B3	Hefei, Anhui	34.9	1–2	SRS4609184	51.9	99.57	82,013	4	47,211	4,133	29,503	1,166	2.11
B8	Hefei, Anhui	235.9	1–1	SRS4609783	61.0	99.68	93,966	3	52,177	4,824	35,635	1,330	2.42
B9	Hefei, Anhui	4034.9	1–1	SRS4610521	67.0	99.61	65,091	2	37,444	3,377	23,376	894	1.68
C10	Nanning, Guangxi	162.1	1–2	SRS4610514	58.6	99.54	127,726	13	69,651	6,654	49,604	1,817	3.29
D2	Zhengzhou, Henan	13.0	1–1	SRS4610558	51.6	99.62	110,117	8	60,030	5,762	42,736	1,589	2.84
D3	Zhengzhou, Henan	4411.0	1–2	SRS4610571	52.0	99.59	108,308	10	59,104	5,576	42,082	1,546	2.79
E3	Chongming, Shanghai	51.5	1–2	SRS4610652	54.0	99.54	37,244	2	22,217	1,957	12,516	554	0.96
E7	Chongming, Shanghai	22.2	1–2	SRS4610661	56.0	99.64	67,654	2	38,662	3,523	24,475	994	1.74
F5	Wuhan, Hubei	0.7	1–1	SRS4610679	55.7	99.63	83,632	50	37,809	4,637	40,101	1,085	2.16
G7	Shenyang, Liaoning	2185.6	1–1	SRS4614286	51.4	99.64	110,239	9	60,349	5,740	42,271	1,879	2.84
H10	Hanzhou, Shanxi	25.2	1–1	SRS4616162	67.4	99.67	95,493	7	53,246	4,866	36,017	1,364	2.46
H2	Hanzhou, Shanxi	32.6	1–1	SRS4614328	60.4	99.68	86,419	3	48,727	4,409	32,054	1,229	2.23
H3	Hanzhou, Shanxi	0.5	1–1	SRS4614441	88.5	99.64	62,395	2	36,904	3,189	21,412	890	1.61
H9	Hanzhou, Shanxi	0.5	1–1	SRS4614451	57.2	99.63	73,988	18	33,884	4,051	35,087	966	1.91
I10	Nanchang, Jiangxi	3.6	1–1	SRS4730442	50.9	99.59	67,914	2	39,671	3,488	23,797	958	1.75
I4	Nanchang, Jiangxi	0.2	1–1	SRS4619648	55.4	99.63	111,259	17	61,031	5,733	42,888	1,607	2.87
I5	Nanchang, Jiangxi	5.8	1–2	SRS4620145	55.3	99.59	66,579	1	38,833	3,381	23,443	922	1.72
J10	Fuzhou, Fujian	0.7	1–2	SRS4730550	55.9	99.65	44,342	0	27,216	2,262	14,211	653	1.14
J8	Fuzhou, Fujian	2.0	1–1	SRS4730448	53.2	99.69	109,903	15	59,785	5,709	42,832	1,577	2.83
K5	Chendou, Sichuan	2124.7	1–2	SRS4730552	52.5	99.68	110,246	9	60,196	5,710	42,802	1,538	2.84
K9	Chendou, Sichuan	3641.9	1–1	SRS4730720	54.3	99.62	97,947	6	54,838	5,061	36,635	1,413	2.53
L7	Yixing, Jiangsu	4.2	1–1	SRS4733465	55.2	99.58	80,588	38	36,898	4,446	38,185	1,059	2.08
M1	Xiangyang, Hubei	477.8	1–2	SRS4733467	57.4	99.69	39,527	0	24,975	1,990	12,008	554	1.02
M2	Xiangyang, Hubei	2.5	1–1	SRS4733602	58.9	99.63	111,342	16	60,834	5,743	43,157	1,608	2.87
M5	Xiangyang, Hubei	1.2	1–2	SRS4733641	53.9	99.71	111,362	9	62,009	5,750	42,009	1,594	2.87
M7	Xiangyang, Hubei	850.0	1–2	SRS4733714	51.9	99.72	81,808	41	37,087	4,430	39,204	1,087	2.11
M9	Xiangyang, Hubei	5905.0	1–2	SRS4733719	67.8	99.62	88,648	5	50,112	4,480	32,848	1,208	2.29
N4	Taizhou, Jiangsu	19.2	1–2	SRS4733770	67.6	99.63	6,712	0	3,219	175	3,226	92	0.17
N5	Taizhou, Jiangsu	5900.3	1–1	SRS4733771	51.1	99.61	16,341	0	10,587	836	4,707	211	0.42
O4	Chaohu, Anhui	873.9	1–2	SRS4733779	54.3	99.56	59,881	1	35,220	3,087	20,735	839	1.54
O5	Chaohu, Anhui	268.4	1–2	SRS4734220	52.3	99.65	98,841	7	55,162	5,055	37,255	1,369	2.55
O7	Chaohu, Anhui	4103.6	1–2	SRS4734283	56.9	99.68	30,610	2	19,207	1,604	9,351	448	0.79
P3	Guangzhou, Guangdong	48.0	1–2	SRS4734256	59.0	99.71	80,842	38	36,395	4,360	39,013	1,074	2.09
P6	Guangzhou, Guangdong	7.0	1–1	SRS4735031	74.3	99.68	96,245	8	53,634	4,857	36,376	1,378	2.48
P7	Guangzhou, Guangdong	2.0	1–1	SRS4735067	54.5	99.66	110,521	14	60,390	5,702	42,839	1,590	2.85
R10	Tai’an, Shandong	699.8	1–1	SRS4738028	50.9	99.73	112,108	13	61,274	5,836	43,390	1,608	2.89
R7	Tai’an, Shandong	542.4	1–2	SRS4735418	53.3	99.63	111,504	13	61,557	5,779	42,598	1,570	2.88
R8	Tai’an, Shandong	6.8	1–1	SRS4735417	54.2	99.71	113,036	10	62,095	5,870	43,432	1,639	2.91
S1	Baoshan, Yunnan	2.8	1–1	SRS4738030	51.0	99.69	83,204	43	37,735	4,602	39,790	1,077	2.17
S3	Baoshan, Yunnan	3001.7	1–2	SRS4740220	65.3	99.58	107,949	13	59,683	5,547	41,207	1,512	2.78
S5	Baoshan, Yunnan	2161.2	1–2	SRS4743831	67.5	99.54	108,594	19	60,050	5,577	41,462	1,505	2.80
S9	Baoshan, Yunnan	2554.5	1–2	SRS4743887	58.7	99.59	109,702	8	60,845	5,675	41,627	1,555	2.83
T3	Yongchuan, Chongqing	0.6	1–1	SRS4748998	56.3	99.61	112,509	12	61,654	5,874	43,357	1,624	2.90
T7	Yongchuan, Chongqing	5.0	1–1	SRS4749290	71.5	99.65	101,781	5	57,462	5,180	37,700	1,439	2.62
T8	Yongchuan, Chongqing	229.3	1–2	SRS4749292	54.5	99.59	111,741	12	61,388	5,726	43,036	1,591	2.88
T9	Yongchuan, Chongqing	3.7	1–1	SRS4749298	56.6	99.67	112,645	9	61,989	5,834	43,187	1,635	2.90
U7	Kaili, Guizhou	3059.6	1–2	SRS4749303	51.1	99.68	109,952	15	60,642	5,664	42,101	1,545	2.84
U8	Kaili, Guizhou	2799.4	1–1	SRS4749553	51.4	99.61	83,413	44	37,736	4,583	40,012	1,082	2.15
V10	Ha’erbing, Heilongjiang	1016.9	1–1	SRS4751061	55.3	99.66	115,168	12	62,995	6,001	44,503	1,669	2.97
V4	Ha’erbing, Heilongjiang	2.9	1–1	SRS4749363	53.9	99.73	108,827	9	60,445	5,612	41,211	1,559	2.81
V6	Ha’erbing, Heilongjiang	1.7	1–1	SRS4749565	50.9	99.59	106,965	3	59,018	5,501	40,906	1,540	2.76
V8	Ha’erbing, Heilongjiang	1.3	1–1	SRS4749564	65.0	99.62	110,936	10	60,978	5,712	42,647	1,599	2.86
W1	Jinan, Shandong	290.9	1–2	SRS4751062	60.7	99.57	11,299	0	6,298	384	4,461	156	0.29
W3	Jinan, Shandong	27.0	1–1	SRS4751209	78.1	99.62	118,235	15	64,315	6,190	46,012	1,718	3.05
W5	Jinan, Shandong	10.3	1–1	SRS4751211	68.5	99.59	85,669	60	38,748	4,733	41,082	1,106	2.21
X4	Tonghua, Jilin	1076.4	1–1	SRS4751212	55.1	99.54	112,319	14	61,668	5,828	43,197	1,626	2.90
X7	Tonghua, Jilin	3.9	1–1	SRS4751219	60.1	99.61	113,963	9	62,820	5,952	43,537	1,654	2.94
X8	Tonghua, Jilin	0.2	1–1	SRS4751488	61.3	99.58	114,379	12	62,647	5,962	44,127	1,643	2.95
S1Fp	Jingzhou, Hubei	0.4	1–1	SRS4740097	70.5	99.57	36,470	3	16,936	1,971	17,061	502	0.94
S2Fp	Xuancheng, Anhui	77.7	1–1	SRS4739228	58.2	99.61	84,499	56	38,166	4,657	40,578	1,098	2.18
S8Fp	Yongkang, Zhejiang	688.7	1–2	SRS4744290	67.8	99.54	84,324	69	38,027	4,580	40,618	1,099	2.18
S10Fp	Changsha, Hunan	6.6	1–1	SRS4748962	62.2	99.73	107,408	11	59,893	5,484	40,530	1,501	2.77
S11Fp	Nanyang, Henan	1.1	1–2	SRS4748961	66.8	99.65	52,033	3	30,502	2,717	18,107	707	1.34
S1Fv	Ha’erbing, Heilongjiang	37.4	1–1	SRS4738501	53.0	99.58	112,345	8	61,380	5,781	43,562	1,622	2.90

### Determination of fumonisin B1 production and fungal biomass

To investigate FB1 content, *F. proliferatum* strains were inoculated on cracked rice kernels or defined liquid (DL, 10 mM glutamine, 22 mM KH_2_PO_4_, 2.5 mM MgSO_4_, 85 mM NaCl, 117 mM sucrose, pH 5.9) media as described with some modification (Shim and Woloshuk, 1999). FB1 production was quantified by high-performance liquid chromatography–tandem mass spectrometry (HPLC–MS/MS) (Li et al., 2012). Briefly, samples were extracted with acetonitrile: water (1:1, v/v) and separated on HPLC (Thermo Fisher Scientific Waltham, MA, United States) equipped with Zorbax Extend-C18 column (100 mm × 2.1 mm, 3.5 μm). Methanol–water–formic acid (75:25:0.2, v/v/v) was used as the mobile phases at flow rate of 0.2 mL/min. The settings were used for MS/MS detection equipped with electrospray ionization (ESI) mode as follows: capillary voltage, 3,500 V; source temperature, 120°C; desolvation temperature, 350°C; and flow rate of desolvation gas, 600 L/h. Ergosterol content was measured as quantitative assessment of fungal growth with HPLC as described previously (Kim and Woloshuk, 2008). Samples were extracted overnight in chloroform: methanol (2:1, v/v). After centrifugation, the supernatants were filtered and injected into HPLC system with Eclipse XDB-C18 column (4.6 mm × 250 mm, 5 μm) and UV detector (Agilent Technologies, Inc., Santa Clara, CA, United States) set to monitor at 282 nm using 100% methanol as the mobile phases at a flow rate of 1.0 mL/min. Finally, FB1 content per fungal biomass was evaluated by the ratio of FB1 production and ergosterol content.

### Genome resequencing and variant calling

Genomic DNA of *F. proliferatum* was extracted from the mycelium using DNeasy Plant Mini Kit (Qiagen, Hilden, Germany) following the manufacturer’s instructions. Paired-end (PE) libraries with insert fragment around 350 bp were constructed and sequenced by Illumina Hiseq4000 platform. Library construction and sequencing were performed at the Beijing Novogene Bioinformatics Technology Co., Ltd., Beijing, China. Raw sequences were pre-processed by the removal of sequencing adapters and low-quality reads by Fastq-mcf with default parameters. Filtered sequencing reads were aligned to the reference genome of *F. proliferatum* strain Fp9 (GenBank accession no. WKFO00000000) using BWA v0.7.8 (Li and Durbin, 2010), and *de novo* genome assembly of the reference strain was previously performed (Wang L. et al., 2022). Spurious reads were filtered with SAMtools v0.1.19 (Li, 2011). Local realignment, duplicate marking and base quality recalibration were further processed using Picard v1.88.^1^ Variant calling for single nucleotide polymorphism (SNPs) and insertions and deletions (Indels) were calculated and identified by GATK v3.6.0 (McKenna et al., 2010) with default parameters. HaplotypeCaller and GenotypeGVCFs, followed by hard filtering with VariantFiltration (generic filter recommendations of GATK plus DP > 200.0, DP < 10.0). All the SNPs retrieved from sequencing reads were mapped to the reference genomes and functionally annotated with SnpEff program (Cingolani et al., 2012).

The genes of *F. proliferatum* strain Fp9 were originally identified through homology searches of *F. proliferatum* strain ET1 sequence (GenBank accession no. FJOF00000000). The SNPs were categorized as occurring in exonic regions (overlapping with a coding exon), intronic regions (overlapping with an intron), splicing sites (within 2 bp of a splicing junction) or intergenic regions. Based on the putative effects of SNPs, the genes were classified as follows, ‘‘non-functional genes’’ with loss of the function, ‘‘modified genes’’ with change of one or few amino acid sequences without major disruption of protein function, ‘‘conserved genes’’ with only change of nucleotide but no change of amino composition, and ‘‘highly conserved genes’’ with no variant in nucleotide sequence. Multigene families were chosen to analyze variants of SNPs. Among them, putative carbohydrate-active enzymes (CAZymes) were identified and classified with the CAZyme database.^2^ Cytochrome P450 monooxygenases (CYPs) were predicted with the fungal cytochrome P450 database.^3^ The homologs of known pathogen-host interaction (PHI-base) genes were predicted using the PHI database.^4^ Secreted proteins with N-terminal signal peptides were predicted by SignalP 5.0 (Almagro et al., 2019). Membrane transporters were predicted by Phobius^5^ and TMHMM (Krogh et al., 2001), and classified based on the fungal transcription factor database.^6^ The annotation of biosynthesis genes of secondary-metabolite (SM) were performed using antiSMURF 4.0 with the Hidden Markov Model (Blin et al., 2017).

### Phylogenetic analysis and population structure

Differences of pairwise SNPs were used to calculate the genetic distances among strains of *F. proliferatum*. The phylogenetic tree was constructed by neighbor-joining method using MEGA 7.0 program (Kumar et al., 2016) based on the genetic differences of strains with 1,000 bootstrap replicates. According to the geographical origin of the strains, three groups including nine strains (G7, X4, X7, X8, V4, V6, V8, V10, and S1Fv), ten strains (A1, A2, A3, E3, E7, N4, N5, J8, J10, and S8Fp) and six strains (C10, K5, U7, S3, S5, and S9) were defined as northeast population, eastern population, and southern population, respectively. Nucleotide diversity (π) within populations, as well as genetic differentiation (fixation index, *F*_*ST*_) and gene flow (*Nm*) between populations were calculated by VCFtools v.0.1.14 (Danecek et al., 2011) with a 50-kb slide window. Population structure was performed using STRUCTURE v.2.3.4 (Pritchard et al., 2000) with the admixture model for the most likely number of clusters (*K*) ranging from 2 to 5. The length of burn-in period and the number of Monte Carlo Markov Chain (MCMC) replications after burn-in were set to 50,000 and 100,000, respectively.

### Genome-wide association study

FB1 production of *F. proliferatum* strains were used as a phenotype for the association analysis. The genome-wide association study (GWAS) was performed by linear regression analysis using PLINK v1.90 (Purcell et al., 2007) with the following parameters: minor allele frequency (>0.05), proportion of missing genotypes (<0.05) and the Hardy-Weinberg equilibrium (> 1e^–5^). *P*-value of each site was estimated and graphically displayed using Haploview v3.2 (Barrett et al., 2005). Raw *p*-values were adjusted for multiple testing with Benjamini-Hochberg correction to control false discovery rate. Sequences for predicted genes at associated loci were retrieved from the reference genome of *F. proliferatum*. Genes with *Q*-value (adjusted *p*-value < 0.05) as cut-off were considered for significance. The regional plots were generated using the *asplot* function in the R package “gap” (Zhao, 2007). The functional annotations were performed on the annotated genes with Trinotate pipeline (Haas et al., 2013).

### Gene deletion and complementation

Deletion mutants of target genes were constructed by homologous recombination. For each gene, upstream and downstream flanking sequences were amplified with the primer pairs 5F/5R and 3F/3R, then cloned into the pFGL821 vector with hygromycin resistance cassette (*hph*). The deletion cassettes were transformed into protoplasts of wild-type strain Fp9 using the polyethylene glycol (PEG) method as previously described (Sun et al., 2019a). Positive transformants were screened with 100 μg/ml hygromycin B (Calbiochem, LaJolla, CA, United States) as selection marker, and verified using PCR with the primer pairs P1/P2 and P3/P4 to confirm gene replacement events. The single copy mutants were checked by the real-time genomic PCR analysis as described before (Wang G. et al., 2022). For complementation assays, the target gene with its full-length promoter region was amplified with primer pairs P5/P6 and co-transformed with *Xho*I-digested plasmid pDL2 into yeast strain XK1-25. The fusion constructs rescued from Trp^+^ yeast transformants were confirmed by sequencing and transformed into the respective knockout mutant as described (Yin et al., 2020). Transformants of complementation were screened with 100 μg/ml geneticin (Sigma, St. Louis, MO, United States) and were identified using primer pairs P5/P6. Primers used for fragments amplification were listed in Supplementary Table 1.

### RNA extraction and relative gene expression analysis

Total RNA was isolated from mycelia of *F. proliferatum* using Trizol reagent (Invitrogen, Carlsbad, CA, United States). RNA quantity was measured on the NanoDrop 2000 spectrophotometer (Thermo Fischer Scientific, Waltham, United States). The cDNA was carried out with the ReverTraAce qPCR RT Master Mix with gDNA Remover Kit (Toyobo, Osaka, Japan) following the manufacture’s protocol. Quantitative real-time reverse transcription PCR (qRT-PCR) was performed in a volume of 20 μl containing 100 ng template cDNA, 10 μl THUNDERBIRD SYBR qPCR Mix (Toyobo, Japan), and 200 nM forward and reverse primers. Reactions were performed on ABI 7300 Real-Time System (Applied Biosystems, Foster City, CA, United States). The β-tubulin encoding gene (*TUB2*) was used as the internal control. Relative transcript level of each gene was calculated by the 2^–Δ^ ^Δ^ *^Ct^* method (Livak and Schmittgen, 2001). qRT-PCR assays were repeated with three biological replicates. Primers utilized for qRT-PCR were shown in Supplementary Table 1.

### Virulence assay and infectious growth

To assay virulence, conidia were harvested from 5-day-old YEPD cultures and re-suspended to 3.0 × 10^5^ conidia per ml in sterile distilled water. Rice spikelets of cultivar Xiushui 134 were inoculated with conidial suspension and examined disease symptoms as previously described (Sun et al., 2019b). Typical symptoms of spikelets were observed at 14 days post-inoculation. For assaying infectious growth, glumes of rice were collected from inoculated spikelets at 24 h post-inoculation and photographed under scanning electron microscope. All the infection assays were repeated at least three times.

### Statistical analysis

The data were presented as means and standard deviations with three biological replicates. Statistical analysis was performed using Student’s *t*-test implemented in the SAS software package (SAS Institute). The level of *p* < 0.05 was considered statistically significant.

## Results

### Genome sequencing and variation discovery

The entire collection of 67 *F. proliferatum* strains covered the regions with frequent outbreaks of RSRD (Figure 1 and Table 1). To investigate the genomic variability of 67 strains, we carried out the whole-genome resequencing using Illumina platform. Sequencing data was submitted to NCBI Short Read Archive (SRA) under the BioProject PRJNA517364 (Table 1). Approximately 500 million PE reads of the 67 sequenced strains were aligned to the reference genome of strain Fp9. The effective mapped read depth ranged from 50- to 100-fold coverage, and the average genome coverage was 99.63%. A total of 5,908,467 SNPs and 883 Indels were observed at non-redundant polymorphic sites (Table 1). Out of these, 3,206,711 variants were located in exonic regions, 307,113 in intronic regions, 2,311,110 in non-genic regions, and 85,426 in splice sites. The average of SNP density was 2.3 per kb in the whole genome of *F. proliferatum*. A little more than half of the SNPs (59.45%) were located in coding domains, with the number of synonymous SNPs amounting to 114,185 vs. 53,401 of that of non-synonymous SNPs (Supplementary Figure 1A). Genes were classified based on functional annotation of variants, and the majority (81.8%) of them were regarded as “modified genes” with non-synonymous effects (Supplementary Figure 1B). The “modified genes” were overrepresented in categories of CAZymes, CYPs, PHI-base, secreted proteins, membrane transporters and SM-encoding genes (Supplementary Figure 2).

### Population genetic structure

**FIGURE 2:**
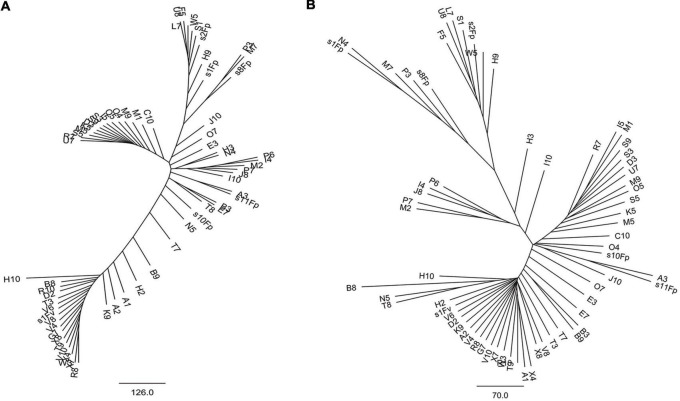
Radial neighbor-joining phylogenetic tree of genetic diversity within *Fusarium proliferatum*. **(A)** Genomic phylogeny determined based on SNP differences using whole-genome alignments of 67 strains to the reference strain Fp9. **(B)** Genomic phylogeny constructed based on SNP differences using *FUM*-cluster alignments of 67 strains to the reference strain Fp9. Branch lengths were proportional to the number of segregating sites that differentiated each pair of strains.

**FIGURE 3:**
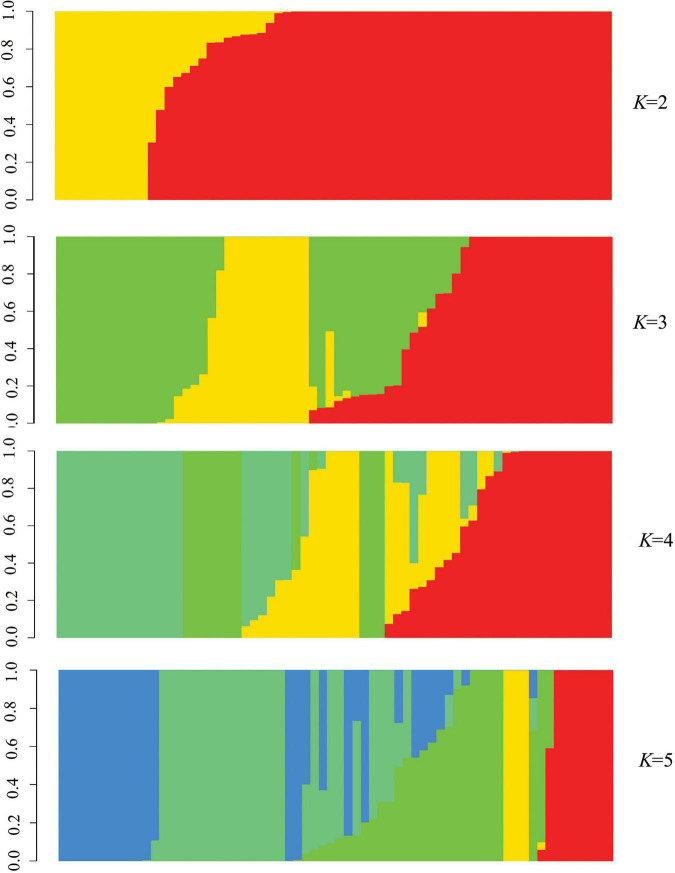
Population structure of *Fusarium proliferatum* strains. Each strain was represented by a vertical bar. Each color represented one ancestral population, and the length of each colored segment in vertical bar represented the proportion contributed by ancestral populations.

Phylogenetic tree in radial pattern was constructed based on the genome-wide SNPs of *F. proliferatum* strains using the neighbor-joining method, which indicated that the strains were genetically independent of each other and not congruent with geographical distribution of the strains (Figure 2A). With minor exceptions, the phylogenetic relationship of strains based on genome-wide SNPs was inconsistent with the characteristics revealed by SNPs variations of *FUM* gene cluster (*R* = 0.007, *p* = 0.957) (Figure 2B). We investigated the genetic structure of three geographical populations. The eastern population of *F. proliferatum* exhibited the highest heterogeneity as indicated by nucleotide diversity (π = 0.213%) relative to that of southern population (π = 0.064%) and northeastern population (π = 0.047%). The eastern population showed low levels of genetic differentiation and moderate levels of gene flow against southern population (*F*_*ST*_ = 0.171, *Nm* = 0.962) and northeastern population (*F*_*ST*_ = 0.178, *Nm* = 0.908), respectively (Table 2). Genetic differentiation between northeastern and southern populations was fairly high (*F*_*ST*_ = 0.334) due to restricted gene flow (*Nm* = 0.248). Structure analysis indicated that *F. proliferatum* population was optimally partitioned with two ancestral components (equivalent to *K* = 2) (Figure 3). The genetic admixture at the individual level (*K* ranging from 3 to 5) revealed the significant signature of within-species introgression in recent evolutionary history. The sexual reproduction of the ascomycetes is determined by MAT loci, and an intact MAT1 locus contains one of the two idiomorphs typical for a heterothallic bipolar mating system. We observed that the frequency (59.7%) of MAT1-1 idiomorph predominated over that of MAT1-2 idiomorph within *F. proliferatum* population (Table 1). Two idiomorphs at the MAT1 locus were identified in the eastern and southern populations, supporting the expectation of footprints of sexual development. Conversely, only single MAT1-1 idiomorph was found in the northeastern population, showing the probability of clonal reproduction during the process of population establishment.

**TABLE 2:** Pairwise comparisons of gene flow (*Nm*, above diagonal) and genetic differentiation (*F*_*ST*_, below diagonal) of *Fusarium proliferatum* populations.

	Northeast population	Eastern population	Southern population
Northeastern population		0.908	0.248
Eastern population	0.178		0.962
Southern population	0.334	0.171	

### Genetic elements associated with fumonisins production

**FIGURE 4:**
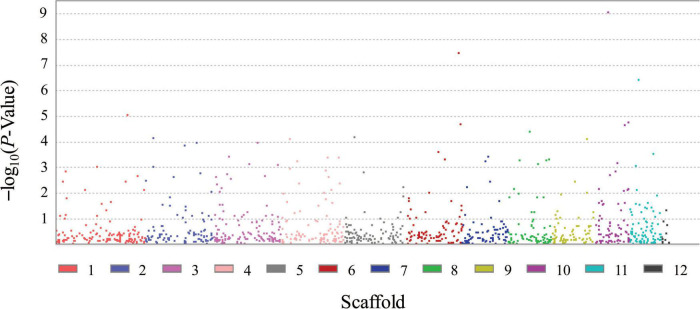
Genomic polymorphisms associated with FB1 production amongst 67 strains of *Fusarium proliferatum*.

FB1 amounts of 67 strains of *F. proliferatum* were determined with rice kernels, ranging from 6.0 to 390,790.9 mg/kg with a mean amount of 46,187.2 mg/kg. Ergosterol was used as an indicator of the fungal biomass. There was the notable difference for relative ratios of FB1 production and ergosterol content among the strains of *F. proliferatum* (Table 1). GWAS was performed to investigate genetic variants associated with phenotypic variation of FB1 production. A total of 121 SNPs was found to be significantly associated with variation of FB1 content per unit of fungal biomass (Figure 4 and Supplementary Table 2). The identified associations were located in 85 candidate genes, and the majority (56.5%) of them were implicated the exon regions. The top 35 candidate genes significantly associated with FB1 production were depicted in Table 3. Functional enrichment analysis with GO term indicated that the genic SNPs were significantly associated with oxidation-reduction processes (GO:0016705, GO:0016491), transmembrane transport (GO:0055085), carbohydrate metabolism (GO:0005975), amine metabolism (GO:0009308), lipid metabolic process (GO:0006629) and chitin biosynthetic process (GO:0006031). Subsequently, we selected five associations in coding regions of genes with FB1 production to validate their functions in the reference strain Fp9.

**TABLE 3:** Top 35 candidate genes significantly associated with FB1 production in *Fusarium proliferatum.*

SNP ID	-log_10_*P*	Location	Gene ontology	Annotation
10:978249	9.07	Intron	GO:0055085; GO:0022857	Nicotinamide mononucleotide permease
6:3814483	7.49	Extron	NONE	Unknown protein
11:704266	6.43	Extron	GO:0055085; GO:0022857; GO:0016020	GABA permease
1:5104401	5.08	Intron	GO:0006355; GO:0000981; GO:0008270	Unknown protein
10:2416760	4.77	Extron	GO:0000160; GO:0007165; GO:0016310; GO:0055085; GO:0000155; GO:0022857; GO:0016772; GO:0016021	Hypothetical protein
10:2185626	4.67	Intron	GO:0000160; GO:0007165; GO:0016310; GO:0055085; GO:0000155; GO:0022857; GO:0016772; GO:0016021	Hypothetical protein
8:1551092	4.43	Extron	GO:0004100; GO:0016758	Chitin synthase D
5:763640	4.20	Extron	NONE	ISWI protein
4:649480	4.13	Extron	NONE	Hypothetical protein
3:3181193	4.00	Extron	NONE	DUF domain protein
2:2827389	3.88	Extron	NONE	Hypothetical protein
6:2347812	3.63	Extron	GO:0006637; GO:0047617	Acyl-CoA thiolesterase
3:1088369	3.45	Extron	GO:0016020	Stomatin family protein
4:3363019	3.42	Extron	GO:0004497; GO:0020037; GO:0016705; GO:0005506	Cytochrome P450
6:2821397	3.33	Extron	NONE	Hypothetical protein
8:845338	3.28	Extron	NONE	Hypothetical protein
8:2772117	3.28	Extron	GO:0050660; GO:0071949; GO:0016491	6-hydroxy-d-nicotine oxidase
7:1643803	3.24	Extron	NONE	Nucleolar complex-associated protein
10:1657227	3.18	Extron	GO:0005975; GO:0055085; GO:0004553; GO:0022857	Beta-glucosidase
8:2182492	3.16	Extron	GO:0007165	Unknown protein
11:467792	3.07	Extron	NONE	Unknown protein
4:633741	2.98	Extron	GO:0003824	Enoyl-CoA hydratase
4:3219957	2.88	Extron	GO:0005515	MFS transporter
1:639420	2.85	Extron	GO:0055085; GO:0022857; GO:0016020; GO:0016021	Unknown protein
2:3915482	2.77	Extron	GO:0020037; GO:0005506; GO:0016705; GO:0004497; GO:0051536; GO:0051537; GO:0016491	Cytochrome P450
3:962262	2.76	Extron	GO:0055085; GO:0005524; GO:0140359; GO:0042626; GO:0016020; GO:0016021	ABC1 transport protein
10:1076521	2.70	Intron	GO:0005975; GO:0003824	Transaldolase B
3:3700605	2.68	Extron	GO:0003723; GO:0003676	KRR1 protein
4:3403391	2.65	Extron	NONE	Beta transducin-like protein
3:224842	2.64	Extron	GO:0016787	Unknown protein
2:35602	2.52	Extron	GO:0008652; GO:0004072	Aspartate kinase
1:491549	2.45	Extron	GO:0071985; GO:0005515	palA protein
9:1619685	2.45	Extron	GO:0006351; GO:0003677; GO:0003899; GO:0008270	DNA-directed RNA polymerase I
4:1336420	2.40	Extron	GO:0005515	Anaphase control protein
4:4196263	2.38	Extron	NONE	Unknown protein

### Examination of possible genetic elements from genome-wide association study

**FIGURE 5:**
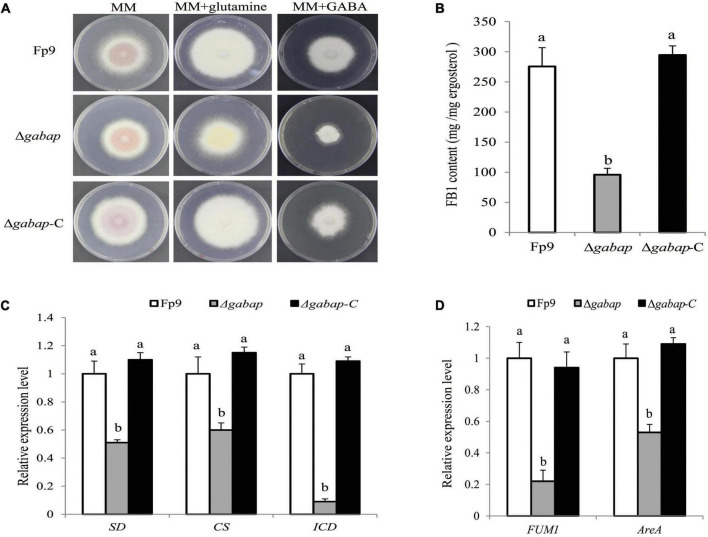
Impact of GABAP on the TCA cycle and FB1 production. **(A)** Five-day-old colony of Δ*gabap* mutant grown on minimal media (MM) containing 30 mM glutamine or GABA as the sole nitrogen source. **(B)** FB1 production of Δ*gabap* cultured with 15-day-old rice kernels. **(C)** The expression levels of *SD* encoding succinate dehydrogenase, *CS* encoding citrate synthase and *ICD* encoding isocitric dehydrogenase of Δ*gabap* grown in YEPD media for 72 h before switching to DL cultures for 3 h. Its relative expression level in wild-type strain Fp9 was arbitrarily set to 1. Different letters on bars indicated a significant difference (*p* < 0.05) with three biological replicates. **(D)** The expression levels of *FUM1* and *AreA* genes of Δ*gabap* grown in YEPD media for 72 h before switching to DL cultures for 48 h. Its relative expression level in wild-type strain Fp9 was arbitrarily set to 1.

The γ-aminobutyric acid (GABA) shunt is a bypass of tricarboxylic acid (TCA) cycle. A strongest association (11:704266) of the gene encoding GABA permease (GABAP) was found to be involved in FB1 production (Table 3). Compared with wide type, the growth morphology of deletion mutant Δ*gabap* appeared unaffected in MM media (Figure 5A). While GABA was supplied as the sole nitrogen source rather than nitrate, the vegetative growth of the mutant Δ*gabap* was retarded as expected. The mutant Δ*gabap* produced only 34.7% as much FB1 amount as that of wild type (Figure 5B). The addition of glutamate, the precursor to GABA, had no impact on the growth and sporulation, but down-regulated gene expression of TCA cycle in the mutant Δ*gabap* (Figure 5C). The transcriptional levels of *FUM1* encoding polyketide synthase responsible for fumonisin biosynthesis and nitrogen metabolite activator *AreA* virtually declined in Δ*gabap* (Figure 5D).

**FIGURE 6:**
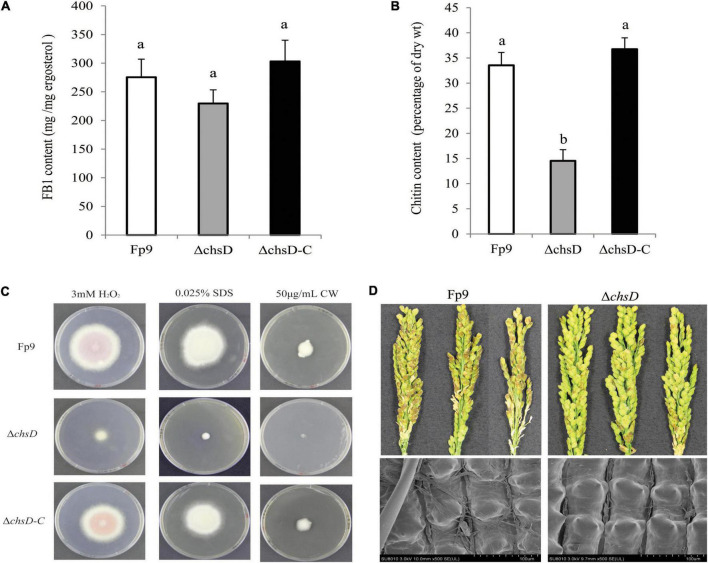
Defects of Δ*chsD* mutant in response to cell wall stress and virulence. **(A)** FB1 production of Δ*chsD* mutant cultured with 15-day-old rice kernels. **(B)** Chitin content of vegetative hyphae of Δ*chsD* harvested from 5-day-old PDA cultures. Different letters on bars indicated a significant difference (*p* < 0.05) with three biological replicates. **(C)** Sensitivity to cell wall perturbing agents of Δ*chsD* on PDA media with 3 mM H_2_O_2_, 0.025% SDS and 50 μg/mL calcofluor white (CW) for 5 days, respectively. **(D)** Disease severity of infected spikelets (upper) and mycelial growth on the outer surface of infected glumes (lower) inoculated with Δ*chsD* strain. Scale bars = 100 μm.

Chitin is an essential component of cell wall in filamentous fungi. A SNP (8:1551092) located in the chitin synthase gene (*chsD*) was detected to be correlated with FB1 production (Table 3). Deletion of *chsD* gene led to reduction, but not significant, of FB1 production by 16.7% (Figure 6A). Compared to wild type, the mutant Δ*chsD* displayed a decrease of 56.7% in chitin content (Figure 6B) and an increase of sensitivity to cell wall stressing agents (Figure 6C). Moreover, pathogenicity assays showed that Δ*chsD* mutant lost virulence on rice (Figure 6D). Perhaps not surprisingly, *chsD* was necessary for cell wall sensitivity and pathogenicity in *F. proliferatum*.

**FIGURE 7:**
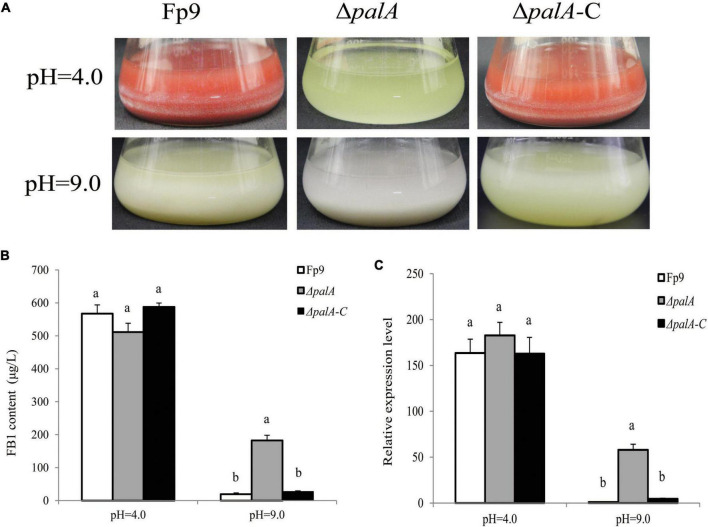
FB1 production activated by PalA in pH-dependent manner. **(A)** Seven-day-old DL cultures of Δ*palA* mutant. **(B)** FB1 production of Δ*palA* grown in YEPD media for 72 h before switching to DL cultures for 7 days at pH = 4.0 and pH = 9.0, respectively. Different letters on bars indicated a significant difference (*p* < 0.05) with three biological replicates. **(C)** The expression levels of *FUM1* gene of Δ*palA* grown in YEPD media for 72 h before switching to DL cultures for 48 h at pH = 4.0 and pH = 9.0, respectively.

PAL signal transduction pathway is known to adapt fungi to sense and respond the changes in the external pH (Peñalva et al., 2008). The pathway relies on the endosomal sorting complex required for transport (ESCRT) complex assembled by PalA and PalB on the endosome membrane, which mediates cytoplasmic proteolysis of transcription factor PacC that activates or represses transcription of acidic- or alkaline-pH responsive genes (Lucena-Agell et al., 2015). We found an associated SNP (1:491549) that was located in the gene encoding PalA (Table 3). FB_1_ amount of disruption mutant Δ*palA* was similar to that of wild type under acidic-inducing conditions, but drastically increased under alkaline-repressing conditions (Figures 7A,B). The transcriptional level of *FUM1* gene was positively correlated with FB1 accumulation (Figure 7C). Accordingly, we concluded that PalA might be involved in FB1 biosynthesis in pH-dependent manner.

**FIGURE 8:**
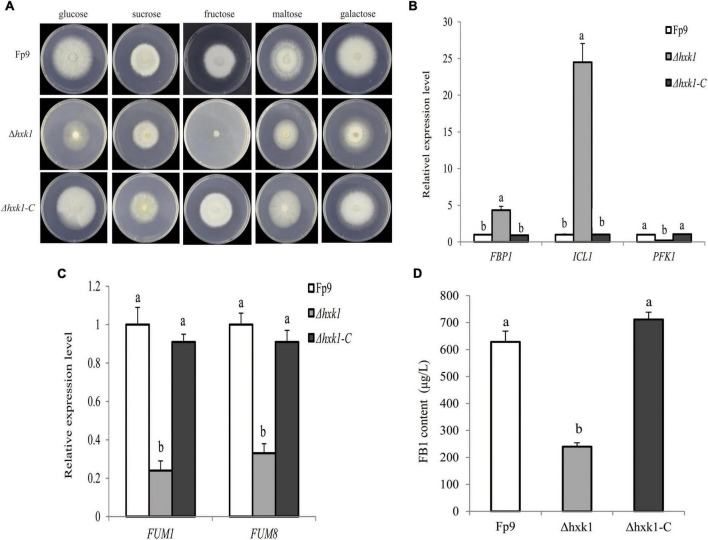
Defects of Δ*hxk1* mutant in fructose assimilation and FB1 production. **(A)** Five-day-old colonies of Δ*hxk1* mutant grown on minimal media (MM) containing glucose, sucrose, fructose, maltose and galactose as the sole carbon source at 55 mM concentration, respectively. **(B)** The expression levels of *FBP1* encoding fructose-1,6-biophosphatas, *ICL1* encoding isocitrate lyase and *PFK1* encoding phosphofructokinase of Δ*hxk1* grown in YEPD media for 72 h before switching to DL cultures for 3 h. Its relative expression level in wild-type strain Fp9 was arbitrarily set to 1. Different letters on bars indicated a significant difference (*p* < 0.05) with three biological replicates. **(C)** The expression levels of *FUM1* and *FUM8* encoding aminotransferase of Δ*hxk1* grown in YEPD media for 72 h before switching to DL cultures for 48 h, respectively. Its relative expression level in wild-type strain Fp9 was arbitrarily set to 1. **(D)** FB1 production of Δ*hxk1* grown in YEPD media for 72 h before switching to DL cultures for 7 days.

Hexokinases (HXK) are known to be involved in carbon catabolism and glucose sensing in fungi (Santangelo, 2006). We found a significant association (6:1695154) of the gene encoding hexokinase1 (HXK1) with FB1 production (Supplementary Table 2). Gene disruption showed that Δ*hxk1* was unable to grow in MM media with fructose as the sole carbon source, and its morphological growth was repaired when glucose was provided (Figure 8A). The transcriptional levels of *FBP1* encoding fructose-1,6-biophosphatase and *ICL1* encoding isocitrate lyase in gluconeogenesis were elevated in mutant Δ*hxk1* relative to wild type, while *PFK1* encoding phosphofructokinase in the glycolytic pathway performed the reverse reaction (Figure 8B). The deletion of HXK1 exhibited obvious reduction in the expression of *FUM* genes and FB1 production (Figures 8C,D). One could simply speculate that the activity of HXK1 was more likely to be responsible for FB1 production *via* sugar metabolism.

**FIGURE 9:**
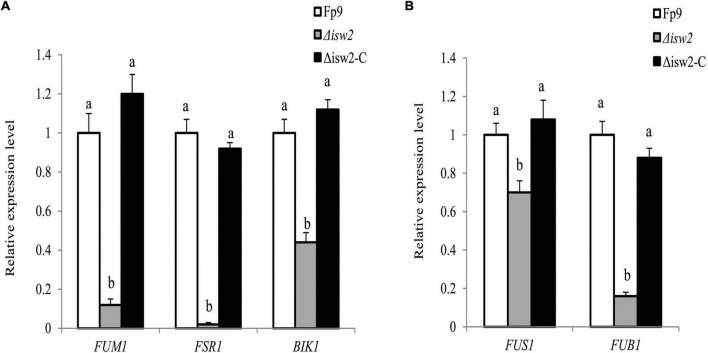
ISW2 affected the expression of SM biosynthetic gene clusters. **(A)** Expression of *FUM1* encoding fumonisin cluster-polyketide synthase, *FSR1* encoding fusarubin cluster-polyketide synthase, and *BIK1* encoding bikaverin cluster-polyketide synthase of Δ*isw2* mutant grown in YEPD media for 72 h before switching to DL cultures for 48 h with 6 mM glutamine as the sole nitrogen source. Its relative expression level in wild-type strain Fp9 was arbitrarily set to 1. Different letters on bars indicated a significant difference (*p* < 0.05) with three biological replicates. **(B)** Expression of *FUS1* encoding fusarin C cluster-polyketide synthase, *FUB1* encoding fusaric acid cluster-polyketide synthase of Δ*isw2* grown in YEPD media for 72 h before switching to DL cultures for 48 h with 60 mM glutamine as the sole nitrogen source. Its relative expression level in wild-type strain Fp9 was arbitrarily set to 1.

The initiation switch (ISWI) is a subfamily of ATP-dependent chromatin remodeling complexes. It can alter nucleosome positioning, catalyze chromatin assembly, and lead to chromosome condensation (Clapier and Cairns, 2012). Of particular interest was the serendipitous observation that a SNP (5:763640) located in the gene encoding ISWI catalytic subunit (ISW2) that was associated with FB1 production (Table 3). Although ISW2 was not required for hyphal growth and pathogenicity, the deletion mutant Δ*isw2* was substantially impaired in FB1 production. Unlike wild type, the lack of ISW2 exhibited down-regulation of the expression levels of SMs cluster genes responsible for fumonisins, fusarubin and bikaverin under nitrogen-starved conditions (Figure 9A), as well as that of the cluster genes responsible for fusarin C and fusaric acid under nitrogen-sufficient conditions (Figure 9B).

## Discussion

In the last decade, RSRD has been one of the most widespread diseases in China, causing severe reduction in rice yield and grains contamination by producing toxic metabolites that are hazardous to humans and livestocks. More importantly, the application of fungicides was not fully operative in controlling accumulation of mycotoxins. Few technologies and approaches are available to prevent fumonisins contamination. Given its economic and public health impacts, an efficient way is to gain the perspective of population biology of *F. proliferatum* before the control is implemented. To the best of our knowledge, this was the first report of the genomic features of *F. proliferatum* population obtained from the major rice-growing region in China (Figure 1 and Table 1). These findings provided new insights into its epidemiologic characteristics and evolutionary processes of this fungus. Such information could contribute to future prospects in designing disease management for RSRD.

The contemporary patterns of pathogen composition and population structure were shaped by historical evolutionary processes over time and space. However, the early history of *F. proliferatum* on cultivated rice, especially the impact of rice domestication on pathogen population, remains still unknown. Here, high level of genetic variation and recombinant mode were found in eastern population of *F. proliferatum* located in the middle and lower valley of the Yangtze River in China, where traditional varieties of *indica* and *japonica* rice were domesticated and co-cultivated with a long history. A hypothetical but straightforward scenario was that the eastern population might be the origin center from where *F. proliferatum* migrate toward the rest of regions, whereas the southern and northeastern populations were the founder populations that may undergo genetic bottlenecks or recent population introduction. There was evidence of gene flow between eastern population and southern or northeastern populations (Table 2), probably as a consequence of human activities like trade and transportation of *F. proliferatum*–contaminated rice seeds.

Intriguingly, southern and northeast populations of *F. proliferatum* showed geographically habitat preferences at the regional scale. Northeast population was represented in areas where *japonica* varieties were cultivated. Conversely, southern population was prevalent in regions where *indica* varieties were grown. The geographical populations of *F. proliferatum* followed the unique evolutionary trajectory, which suggested the signature of rice domestication as the major driving force for population divergence. In *Microbotryum* fungi, species-specific genes corresponded to putative adaptations responding to host-mediated selection (Badouin et al., 2017). Such epidemiological implications, probably accompanied by the host specialization, exemplified that the deployment of resistance cultivars must keep up with co-evolutionary “arm races.” By introducing the genes, such as major resistance genes and defense-responsive or defense-related genes specific for one subspecies into the other subspecies, *F. proliferatum* might confront selection pressures imposed by host resistance that it has never met before.

Sexual reproduction plays the fundamental roles in maintaining the genetic diversity and enhancing the adaptation to environments in fungi. Nonetheless, the stage of teleomorph of *F. proliferatum* has not been well described. One reason for the widespread spread of *F. proliferatum* was its ability to produce vast numbers of mitotically derived asexual spores, namely conidia. Our results supported that heterothallism was likely to occur frequently in the eastern and southern populations demonstrated by existence of two MAT idiomorphs (Table 1). The opposite mating types were needed for the perithecium formation of *F. proliferatum*. Epidemic of RSRD was possibly aggravated by the ability of the pathogen to spread through airborne ascospores. The phenomenon of *F. proliferatum* strains containing both MAT idiomorphs over large regions was congruent to the previous observations of that collected from durum wheat and garlic bulb (Palacios et al., 2015; Gálvez et al., 2017). The evidences implied that there was probability that *F. proliferatum* propagate asexually by conidia and sexually by ascospores allowing both selfing and outcrossing. The strains with strong competition exhibit dominance in geographical areas, which enhanced its potentiality to respond to disease management, e.g., fungicides modes, resistant cultivars, agronomic practices, or even the climate change. In addition, a single clonal lineage of mating type MAT1-1 was detected in northeast population (Table 1). From the practical standpoint, the finding highlighted the need for persistent vigilance of northeast population, to prevent the invasion of MAT1-2 idiomorph that might initiate sexual component to the rice-*F. proliferatum* pathosystem.

On a broader scale, the genome-wide level of polymorphism in *F. proliferatum* was 2.3 variants per kb (Table 1), which was similar to that in wheat powdery mildew *Blumeria graminis* (1.4–2.0 SNPs/kb) (Wicker et al., 2013), larger than blackleg *Leptosphaeria maculans* (0.5 SNPs/kb) (Zander et al., 2013), but much lower than poplar rust *Melampsora larici-populina* (6 SNPs/kb) (Persoons et al., 2014). Naturally, plant pathogens with higher diversity have relative advantages of the ecological fitness. Combining effects of natural selection, sexual recombination and genome-wide polymorphism, *F. proliferatum* was considered as the high-adaptive pathogen with the ability to occupy ecological niches or infect broad range of hosts during fungal population expansion. In this regard, continued surveillance of pathogen will be critical for evaluation of resistant cultivars. Resistance breeding can benefit from the inclusion of representative strains of *F. proliferatum* rather than a single strain when screening resistance varieties to RSRD.

There was the remarkable difference in capability of fumonisins biosynthesis among *F. proliferatum* strains from rice (Table 1). Similar phenotypic differences were observed among *F. proliferatum* strains from asparagus (von Bargen et al., 2009), pineapple (Stêpieñ et al., 2013), pea (Waśkiewicz et al., 2013), and garlic (Gálvez et al., 2017). It has been proved that the regulatory networks for fungal SMs are modulated by hierarchical interconnection with diverse cellular processes from cluster-specific pathways to global transcriptional complexes (Macheleidt et al., 2016). Biogenesis of fumonisins at the levels of pathway steps and their regulation remains a challenging task. There is a knowledge gap in the genetic diversity and fumonisins production capacity of *F. proliferatum*. Several attempts have been made to decipher certain interested genes located outside *FUM* gene cluster, it is not sufficient to elucidate the unexplored biomolecules responsible for fumonisins formation. This work aimed to depict the regulation factors involved in fumonisins production in *F. proliferatum* using GWAS based on whole-genome resequencing. Currently, GWAS is a preferable method for detection of the genetic determinants associated with phenotypic variation for fungal pathogens at species level, such as cold tolerance in *Clonostachys rosea* (Broberg et al., 2018), virulence in *Zymoseptoria tritici* (Hartmann et al., 2017), as well as aggressiveness, deoxynivalenol (DON) production and azole sensitivity in *Fusarium graminearum* (Talas et al., 2016). As far as we know, this is the first study to apply GWAS to link polymorphism in the genome to functional variability associated with fumonisins production among natural populations of *F. proliferatum* strains.

We identified that oxidation-reduction processes (GO:0016705, GO:0016491) were connected with fumonisins biosynthesis in *F. proliferatum* (Table 3). Similarly, the activation roles of cellular redox stress were previously found on aflatoxin biosynthesis in *Aspergillus flavus* (Fountain et al., 2019) and trichothecene production in *F. graminearum* (Montibus et al., 2013). The majority of oxidation, reduction and hydrolysis reactions are performed by CYPs. CYPs enzymes have unique ability to catalyze regio-, chemo-, and stereospecific conversions of lipophilic compounds to hydrophilic derivatives by introducing an oxygen atom. Three members from CYPs (FPRO_06718, FPRO_05147, FPRO_05649) were identified to be related with fumonisins production (Supplementary Table 2). Several CYPs participating in SMs biosynthesis pathways had been characterized in other fungi. Among 26 genes located in aflatoxin biosynthesis gene cluster, *aflG*, *aflQ*, *aflU*, and *aflV* encoded CYPs in *A. flavus* (Yu et al., 2004). In *F. graminearum*, three CYPs (*TRI1*, *TRI4*, and *TRI11*) were responsible for trichothecene biosynthesis, and three CYPs encoded ergosterol biosynthetic enzymes (Shin et al., 2017). In *Verticillium dahliae*, *VdCYP1* had a pronounced effect on 14 kinds of fungal metabolites that likely contributed to pathogenic process (Zhang D. D. et al., 2016). Therefore, it was conceivable that the catalytic regulation of fungal CYPs was essential for cell viability and secondary metabolism.

Transmembrane transport (GO:0055085) was found to be involved in fumonisins production (Table 3). Transporters are responsible for nutrient uptake, metabolite extrusion, multidrug resistance and signal exchange. ATP-binding cassette (ABC) transporters are a superfamily of proteins that use the energy from ATP hydrolysis to transport substances across the cell membrane. Our results showed that fumonisins production was affected by an ABC transport (FPRO_03446) (Supplementary Table 2). This seemed to be the case for *Bacillus cereus* as well because ABC transporters were involved in the biosynthesis of metabolites, such as toxins, antibiotics and siderophores (Gacek-Matthews et al., 2020), but the underlying mechanisms remain unclear. Peptide transporters belonging to the major facilitator superfamily (MFS) transporters use the proton-motive force to translocate di- and tripeptides. Internalized peptides are rapidly hydrolyzed and the resulting amino acids are used for protein synthesis or alternative sources of nitrogen and carbon. We found that a peptide transporter (FPRO_13944) was associated with fumonisins biosynthesis (Supplementary Table 2). In *F. graminearum*, deletion of peptide transporters (FgPTR2A, FgPTR2C, and FgPTR2D) resulted in a sharp increase in DON and zearalenone and a decrease in fusarielin H. The three mycotoxins fell under the regulation of nitrogen regulator AreA because that the transportation activity of peptide transporters was influenced by the quality of the nitrogen source (Droce et al., 2017). Further studies are required to understand the exact nature of how the peptide transporters modulate the genetic regulatory network underlying the SM biosynthesis.

Carbohydrate metabolic process (GO:0005975) was shown the association with fumonisins production in *F. proliferatum* (Table 3). The sources of carbon available were reported to play a critical role in induction of SM biosynthesis, such as fumonisins biosynthesis in *F. verticillioides* (Malapi-Wight et al., 2013), aflatoxin production in *A. flavus* (Fasoyin et al., 2018), ochratoxin A biosynthesis in *Aspergillus ochraceus* (Wang et al., 2020). Glucose is the preferred carbon source for the filamentous fungi. Several genes encoding enzymes (e.g., xylanases, cellulases, and arabinases) required for the use of alternative carbon sources, such as lignocellulose, are repressed by carbon catabolite repression (CCR) when glucose is present. From the GWAS, three significant SNPs were located in genes encoding β-glucosidase (FPRO_14041, FPRO_03671, FPRO_15192) (Supplementary Table 2). β-glucosidase mainly hydrolyze β-glucosidic bonds to release glucose from the non-reducing end of β-glucoologosaccharides or glucosides. This effect was mediated in part by sucrose non-fermenting (SNF1) kinase that was regarded as an important regulator in cellulose degradation and sterigmatocystin biosynthesis in *Podospora anserina* (Li et al., 2020). Several fungal and bacterial β-glucosidases had the abilities to hydrolyze the *Fusarium* mycotoxins, such as DON, nivalenol and HT-2 toxin (Michlmayr et al., 2015). Thus, this would be reasonable that fungi have evolved mechanisms to balance the operation of substrate feeding (enzyme production) and secondary metabolism during life cycle.

Amine metabolic process (GO:0009308) was found to exert influences on fumonisins biosynthesis (Supplementary Table 2). Nitrogen status directly affected the ability of filamentous fungi to biosynthesize SMs (Tudzynski, 2014). Biogenic amines are low molecular weight organic nitrogen compounds in living cells. Amine oxidases catalyze the oxidative deamination of amine substrates to their corresponding aldehydes, including aliphatic and aromatic monoamines, diamines, tertiary amines, polyamines, and amino acids. Amine oxidases were known to be involved in primary amine metabolism, but the effects of amine oxidases on SMs production is rare. Two genes encoding amine oxidase (FPRO_13752, FPRO_06592) were identified to be related to fumonisins production in *F. proliferatum* (Supplementary Table 2). Amine oxidases of *Aspergillus niger* and *Cochliobolus victoriae* were capable of enzymatic conversion of fumonisins and victorin into deaminated and detoxified counterparts, respectively (Garnham et al., 2020; Kessler et al., 2020), which demonstrated relaxed substrate specificity of amine oxidases in mycotoxins biosynthesis.

The *gabap* gene encoding GABA permease was found to be significantly associated with FB1 production (Figure 5B). The *gabap* mutant failed to synthesize FB1 and inactivated the expression of *FUM1* gene responsible for fumonisin biosynthesis. There were several indications for the connectivity between GABA shunt and the regulation of FB1 production. GABA metabolism had been previously described to link MFS transporters that pumped out DON mycotoxin in *F. graminearum* (Wang et al., 2018). The addition of GABA-inducible agmatine promoted the production of starting substrate for DON biosynthesis in *Fusarium asiaticum* (Suzuki et al., 2013). Deletion of *gabap* impaired GABA utilization in *F. proliferatum*, which was partially regulated by the global nitrogen regulator AreA. The mutant Δ*gabap* showed downregulation of the key enzymes of the TCA cycle. Thus, we proposed that GABA as a nitrogen source positively regulated FB1 accumulation in *F. proliferatum*.

We identified a correlation between *chsD* gene encoding chitin synthase and FB1 production (Figure 6A). Disruption of *chsD* resulted in reduced accumulation of chitin and enhanced sensitivity against cell wall perturbing agents. In addition, our study found that chitin synthase played an important role in pathogenicity in *F. proliferatum* as previous studies on *Magnaporthe oryzae* (Kong et al., 2012) and *Botrytis cinerea* (Cui et al., 2013). The reduced FB1 production in the Δ*chsD* deletion mutant further verified the involvement of chitin synthase in aggressiveness since FB1 played an important role in the infection of *F. proliferatum* in rice tissue (Sun et al., 2019a). The effect of *chsD* gene on FB1 production in *F. proliferatum* was similar to that on DON production in *F. graminearum* (Liu et al., 2016). Our results demonstrated that essentiality of chitin biosynthesis in vegetative growth, virulence and FB1 production in *F. proliferatum*, which allow the development of novel inhibitory agents in the control of this pathogen by targeting the chitin synthase.

Fungal microorganisms must be able to sense and respond to ambient pH for mycelial growth, host colonization and toxin production. The ambient pH signaling pathway in filamentous fungi was medicated by six Pal proteins (PalA, PalB, PalC, PalF, PalH, and PalI) and transcription factor PacC (Peñalva et al., 2008). It was clear from previous studies that PacC was a key factor for biosynthesis of various SMs, such as ochratoxin A in *A. ochraceus* (Barda et al., 2020), fumonisins in *F. verticillioides* (Flaherty et al., 2003), dipicolinic acid in *Beauveria bassiana* (Luo et al., 2017), patulin in *Penicillium expansum* (Chen et al., 2018). The activation of PacC by proteolysis required interactions of with PalA and PalB in an alkaline pH dependent manner. In the present study, the *palA* gene was involved in the association of FB1 production (Figure 7B). Genetic disruption of *palA* rendered enhanced FB1 accumulation under external pH alkalinization in *F. proliferatum*. To further characterize the mechanism of the environmental pH-sensing complex on fumonisins, studies are underway to dissect this intriguing phenomenon.

Our findings suggested a relationship between *hxk1* gene encoding hexokinase and FB1 biosynthesis in *F. proliferatum* (Figure 8D). The deletion mutant of *hxk1* failed to grow on fructose and down-regulated the expression level of glycolytic genes. More importantly, Δ*hxk1* produced a dramatically low level of FB1, indicating that *hxk1* was involved in FB1 biosynthesis in *F. proliferatum*. Pyruvate, the end-product of glycolysis, was controlled by hexokinase as the main substrate of acetyl-CoA for the biosynthesis of many SMs (Gao et al., 2016), including fumonisins, aflatoxin, trichothecene, DON, and penicillin. HXK1 had been shown to regulate DON production in *F. graminearum* (Zhang L. et al., 2016) and FB1 production in *F. verticillioides* (Kim et al., 2011). Our observations demonstrated that HXK1 was required for FB1 biosynthesis by establishing genetic link between primary and secondary metabolism in *F. proliferatum*.

ISWI catalytic subunit (ISW2) belonging to a subfamily of chromatin remodeling complexes was associated with FB1 production (Figure 9A). Perhaps the more striking observation was that the effects of ISW2 on biosynthesis of multiple SMs were regulated by nitrogen availability in *F. proliferatum*. The concept of ISW2 triggering SMs biosynthesis in filamentous fungi was almost without precedent. The chromatin remodeling complex RSC1 had been well characterized in *Saccharomyces cerevisiae*, which was required for TOR protein kinase (Yu et al., 2015). TOR signaling pathway is a central regulatory hub that connects nitrogen availability with fungal development and metabolic processes (Dobrenel et al., 2016). Therefore, it was quite likely that ISW2 played an indirect role for secondary metabolism by establishing the genetic link between nutrient-signaling regulation and SMs production.

As noted above, five candidate genes (*gabap*, *chsD*, *palA*, *hxk1*, and *isw2*) from the GWAS that were all required for FB1 production, possibly reflecting pleiotropic effects in coordinating the processes of fumonisins biosynthesis. It was very likely that, in such cases, SM production of filamentous fungi was controlled by a complex regulatory and biosynthesis pathways in a manner related to fungal development or in response to abiotic and biotic stressors. However, it was worthy to note that some associations were poorly annotated with unknown contributions to SM production. Further research should be intensified to identify and validate the physiological functions of the genetic components. The top hit from our association analysis will serve as excellent targets for genetic and biochemical efforts to detoxify or decontaminate mycotoxins of *F. proliferatum*.

## Conclusion

In this study, we firstly characterized the intraspecific genomic variation of *F. proliferatum* populations from rice in China, to highlight rapid evolution shaped by high genetic diversity and sexual recombination, and elucidate selection pressures imposed by host domestication on population structure. These findings provided the evidences that the host-specific selection left signatures of genetic differentiation in *F. proliferatum* populations. Additionally, this work indicated the capability of association mapping to pinpoint genetic determinants involved in fumonisins formation in *F. proliferatum*. The regulatory factors related with secondary metabolism need to be validated for their potential functions. The sustainability of resistant cultivars and fungicides used to control RSRD should be geared toward information on the population characteristics of *F. proliferatum*, which contribute to monitor the influx of pathogen with novel adaptations, as well as discover pharmaceutical chemicals to impact positively on food safety and security.

## Data availability statement

The datasets presented in this study can be found in online repositories. The names of the repository/repositories and accession number(s) can be found at: https://www.ncbi.nlm.nih.gov/bioproject/?term=PRJNA517364.

## Author contributions

LW, ST, and PH contributed to the idea and design of the research. LW, GJ, XW, GS, and LX collected the strains. LW, SG, WHL, WYL, and WL conducted the experiments. QL was responsible for the whole-genome sequencing and analysis work. ZS and SH collected the data and performed statistics. LW wrote the first draft of the manuscript. ST and PH performed the revision and editing of the final manuscript. All authors contributed to revisions of the manuscript, read, and approved the submitted version.

## Footnotes

1
https://github.com/broadinstitute/picard

2
http://www.cazy.org/

3
http://drnelson.uthsc.edu/cytochromeP450.html

4
http://www.phi-base.org/

5
https://phobius.sbc.su.se/

6
http://ftfd.snu.ac.kr/
