# Supplementary material for: Genomic footprints related with adaptation and fumonisins production in Fusarium proliferatum
Source: Front Microbiol. 2022 Sep 21;13:1004454. doi: 10.3389/fmicb.2022.1004454 (PMC9532532; doi:10.3389/fmicb.2022.1004454)
Supplement: Supplementary file 1 [file Data_Sheet_1.docx]

Supplementary Material

# Supplementary Figures and Tables

## Supplementary Figures


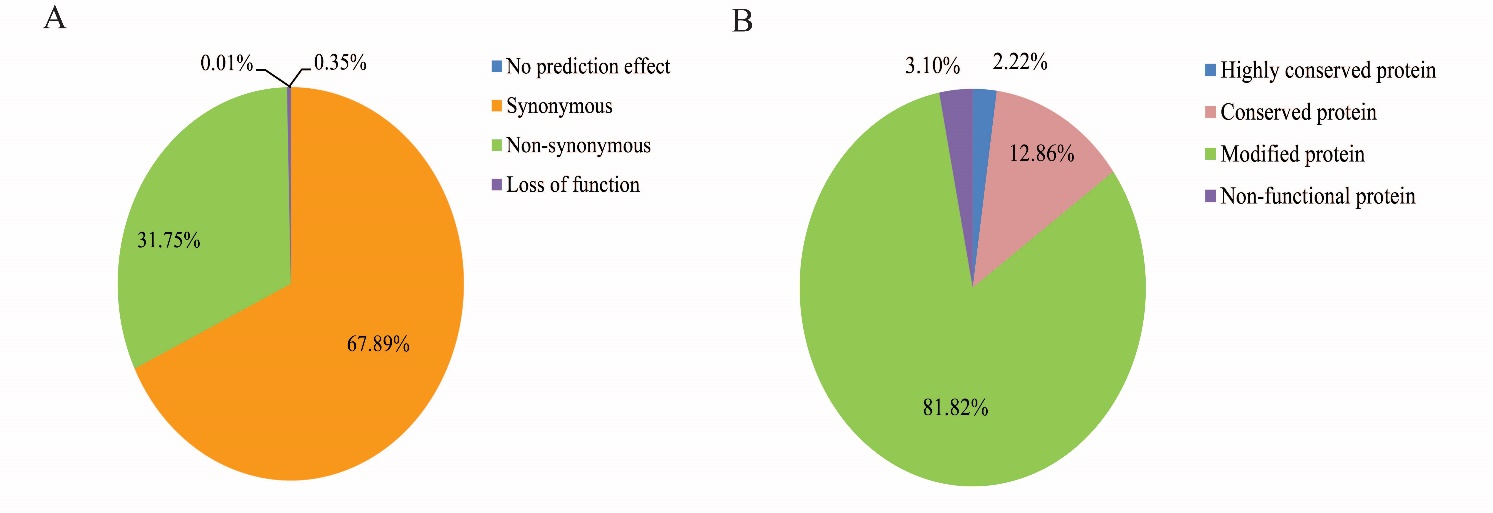


**Supplementary Figure 1** Determination of variants and their predicted effects on genes. **(A)** Classification of variants (n=168,182) in coding sequences based on their features of position and function. The blue (0.01%) represented variants with no prediction effect, the orange (67.89%) represented variants with synonymous SNPs, the green (31.75%) represented variants with non-synonymous SNPs, and the purple (0.35%) represented variants leading to loss of function of the proteins. **(B)** Classification of genes (n=14,054) based on the effects of variants. The blue (2.22%) indicated “highly conserved genes” with no variant in nucleotide sequence, the pink (12.86%) indicated “conserved genes” that had only changes in nucleotides without a change in amino acid composition, the green (81.82%) indicated “modified genes” that changed one or few amino acid composition without disruption of protein function, and the purple (3.10%) indicated “non-functional genes” that contained variants leading to loss of function of the proteins in at least one strain.


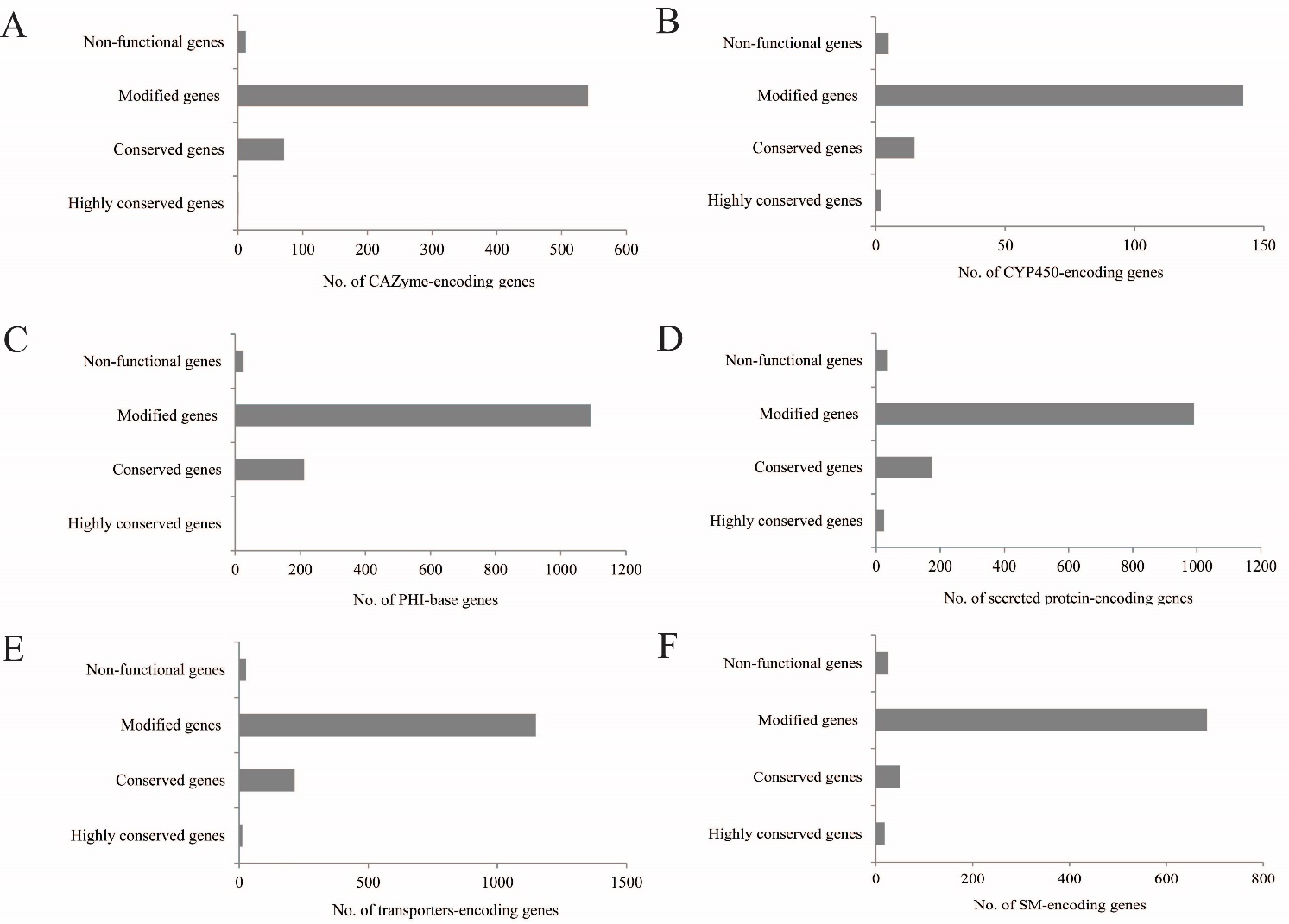


**Supplementary Figure 2** The functional classification of variants in selected gene families. **(A)** The classification of variants for CAZyme-encoding genes (n=625). **(B)** The classification of variants for CYPs-encoding genes (n=164). **(C)** The classification of variants for PHI-base genes (n=1,330). **(D)** The classification of variants for secreted protein-encoding genes (n=1,222). **(E)** The classification of variants for transporters-encoding genes (n=1,402). **(F)** The classification of variants for SM-encoding genes (n=778).

## Supplementary Tables

**Supplementary Table 1** Primers used for deletion, complementation and gene expression

| **Gene Name** | **Gene ID** | **Putative Function** | **Primer name** | **Sequences (5’-3’)** | **purpose** |
| --- | --- | --- | --- | --- | --- |
| *gabap* | FPRO_15550 | GABA permease | gabap-5F | AGAAACTCGAGAATTCGAGGGCTGTCATTTCG | *gabap deletion* |
|  |  |  | gabap-5R | TATACTCGACTCTAGAAGATTTCTGGGCTTGG |  |
|  |  |  | gabap-3F | CGGAACCAGTCGACCTGCAGCAGAAGTGCCCTGAAACG |  |
|  |  |  | gabap-3R | GGCCAGTGCCAAGCTTCCCAACGGAATAGGATGA |  |
|  |  |  | gabap-P1 | AATAATCTATCCGAGTCGTT | verification of *gabap* deletion |
|  |  |  | gabap-P2 | GTGACACCCTGTGAACG |  |
|  |  |  | gabap-P3 | GTGTATCACTGGCAAACTG |  |
|  |  |  | gabap-P4 | ACCAACGCAAGCAAGAT |  |
|  |  |  | gabap-P5 | GTATTTAGTCCGCCTTGTC | *gabap* complementation |
|  |  |  | gabap-P6 | CCACGGTAGTAATGGGTT |  |
| *chsD* | FPRO_11929 | Chitin synthases D | chsD-5F | AGAAACTCGAGAATTCTCAACAGAGGGAGGCG | *chsD deletion* |
|  |  |  | chsD-5R | TAGTGGATCCCCCGGGTACCTGCAGAGCGGCATTAC |  |
|  |  |  | chsD-3F | CGGAACCAGTCGACCTGCAGTAAGGCAATGGGTTAT |  |
|  |  |  | chsD-3R | GGCCAGTGCCAAGCTTTATCTCGCTTGTGGTC |  |
|  |  |  | chsD-P1 | CTGTGACGAGGTTGGGATG | verification of *chsD* deletion |
|  |  |  | chsD-P2 | TGTGAACGGCGGGAGAT |  |
|  |  |  | chsD-P3 | CCGTGGTTGGCTTGTAT |  |
|  |  |  | chsD-P4 | TATTCCGTTTGCTTTCG |  |
|  |  |  | chsD-P5 | CTCCGATTCTACCTTTGC | *chsD* complementation |
|  |  |  | chsD-P6 | TGGACTAACTCACCGACA |  |
| *palA* | FPRO_00198 | pH-response regulator protein | palA-5F | TATGGAGAAACTCGAGAATTCACTCACCCTGCCTAATCTCACAA | *palA deletion* |
|  |  |  | palA-5R | CTAGTGGATCCCCCGGGTACCTGCATACATGGCGAATACGAGT |  |
|  |  |  | palA-3F | CGGAACCAGTCGACCTGCAGGGGGTAGCTTCGTGTCA |  |
|  |  |  | palA-3R | GGCCAGTGCCAAGCTTAGCGTCGTTGTACCAGTT |  |
|  |  |  | palA-P1 | GGGAAGGCAAGGAGTGG | verification of *palA* deletion |
|  |  |  | palA-P2 | TCGGTTTCAGGCAGGTC |  |
|  |  |  | palA-P3 | GCCGTGGTTGGCTTGTA |  |
|  |  |  | palA-P4 | CGCTGGAGGTCTGTATTTG |  |
|  |  |  | palA-P5 | TCACTCGCCGACTAAACC | *palA* complementation |
|  |  |  | palA-P6 | CCAGCGCATCATACAAAA |  |
| *hxk1* | FPRO_09597 | hexokinase | hxk1-5F | CGAGAATTCGAGCTCGGTACCTGAGCATGTCGCAATCCTCG | *hxk1 deletion* |
|  |  |  | hxk1-5R | TTCAATATACTCGACTCTAGATCGGTGTCTGGCGTTTTGG |  |
|  |  |  | hxk1-3F | CGGAACCAGTCGACCTGCAGGGCAGAATGTAAATAGTCCA |  |
|  |  |  | hxk1-3R | GGCCAGTGCCAAGCTTGTTTGAGAAGCCGCACT |  |
|  |  |  | hxk1-P1 | GTAGAGCAACTTGGACCTT | verification of *hxk1* deletion |
|  |  |  | hxk1-P2 | GTGACACCCTGTGAACG |  |
|  |  |  | hxk1-P3 | CCGTGGTTGGCTTGTAT |  |
|  |  |  | hxk1-P4 | CGTTGACTCCGTCTACTCC |  |
|  |  |  | hxk1-P5 | TTTCTTTGACCACCTTTG | *hxk1* complementation |
|  |  |  | hxk1-P6 | CTACTACACCTTGCTCCCT |  |
| *isw2* | FPRO_08500 | ITC1-subunit of Isw2 chromatin remodelling complex | isw2-5F | AGAAACTCGAGAATTCGGCTATGCCTCCTTTCT | *isw2 deletion* |
|  |  |  | isw2-5R | TAGAACTAGTGGATCCCGGTGCTAAGACGAAAT |  |
|  |  |  | isw2-3F | CGGAACCAGTCGACCTGCAGTATCCGTCTCGTATCAA |  |
|  |  |  | isw2-3R | GGCCAGTGCCAAGCTTGTACATGGCGTAAAGG |  |
|  |  |  | isw2-P1 | ACCTTGCTTCTTCACCCTT | verification of *isw2* deletion |
|  |  |  | isw2-P2 | GTCGTCCATCACAGTTTGC |  |
|  |  |  | isw2-P3 | ACGGCAATTTCGATGAT |  |
|  |  |  | isw2-P4 | CTGGGAGCGTGACTTCT |  |
|  |  |  | isw2-P5 | CTGGCAGTGGCATAAGGG | *isw2* complementation |
|  |  |  | isw2-P6 | CGGGTCGATCTATTTATTAGCA |  |
| AreA | FPRO_09627 | nitrogen regulatory protein | qAreA-F | AATCGCAACCTCAAGTCC | qRT-PCR analysis |
|  |  |  | qAreA-R | GGGAGCGTTGTGAATAGC |  |
| *FUM1* | FPRO_13593 | fumonisin polyketide synthase | qFUM1-F | CCAACTCTTCTTCCCTGCTA | qRT-PCR analysis |
|  |  |  | qFUM1-R | CACCCTCTACCTCCCACA |  |
| *FSR1* | FPRO_03539 | fusarubin polyketide synthase | qFSR1-F | CCTCAACAGCGAAACCCT | qRT-PCR analysis |
|  |  |  | qFSR1-R | AAGCCACAAGACTGAGTAAGAG |  |
| *BIK1* | FPRO_07275 | bikaverin polyketide synthase | qBIK1-F | CGTCCACAAACCAGTCCC | qRT-PCR analysis |
|  |  |  | qBIK1-R | TTTAACCAGCAGCCGTAC |  |
| *FUS1* | FPRO_12771 | fusarin C polyketide synthase | qFUS1-F | GAAGGATGTGCTGGTCTTG | qRT-PCR analysis |
|  |  |  | qFUS1-R | CTTGAGGGCAGGGTTTAG |  |
| *FUB1* | FPRO_03870 | fusaric acid polyketide synthase | qFUB1-F | TGATTGCCAATGGATCTCA | qRT-PCR analysis |
|  |  |  | qFUB1-R | TATGCCTGGAGGGTTTGA |  |
| *SD* | FPRO_01659 | succinate dehydrogenase | qSD-F | TCTGACCGTCGCTCCCTT | qRT-PCR analysis |
|  |  |  | qSD-R | CGATGACAACCTGCTGGAA |  |
| *CS* | FPRO_00500 | citrate synthase | qCS-F | TTCTCCTATCAATGGCTCTGT | qRT-PCR analysis |
|  |  |  | qCS-R | TGACCTGCTCAATCTTCTCG |  |
| *ICD* | FPRO_02284 | isocitrate dehydrogenase | qICD-F | GTGCCGCTCTTGTTGGT | qRT-PCR analysis |
|  |  |  | qICD-R | GGTCCTTGCCCTTGATG |  |
| *ICL* | FPRO_08144 | isocitrate lyase | qICL-F | CAGGCGAAGAAGTTGTGG | qRT-PCR analysis |
|  |  |  | qICL-R | CATAGACGGTGTCGAGGTAC |  |
| *FBP1* | FPRO_03384 | fructose-1,6-bisphosphatase | qFBP1-F | CCTCACCCGCTTCCTCACTG | qRT-PCR analysis |
|  |  |  | qFBP1-R | GCGTCGAATGTAGTAGGCAAT |  |
| *PFK1* | FPRO_03195 | 6-phosphofructokinase | qPFK1-F | ACATCCTAGCCGACAAGAG | qRT-PCR analysis |
|  |  |  | qPFK1-R | CAGGGTAGCCAACATTCG |  |
| *TUB2* | FPRO_01113 | β-tubulin | qTUB-F | ACATCCAGACAGCCCTTTGTG | qRT-PCR analysis |
|  |  |  | qTUB-R | AGTTTCCGATGAAGGTCGAAGA |  |

**Supplementary Table 2 Significant associated SNPs (*p*<0.05) with FB1 production in *F. proliferatum***

| **SNP ID** | **-log_10_P** | **Gene ID** | **Location** | **Reference allele** | **Alternative allele** | **Frequency** | **Gene Ontology** | **Annotation** |
| --- | --- | --- | --- | --- | --- | --- | --- | --- |
| 10:978249 | 9.07 | FPRO_14315 | intron | G | A/C | 0.104/0.015 | GO:0055085;GO:0022857 | nicotinamide mononucleotide permease |
| 6:3814483 | 7.49 | FPRO_08908 | extron | G | A/C | 0.090/0.015 | NONE | unknown protein |
| 11:704266 | 6.43 | FPRO_15550 | extron | T | A/C | 0.134/0.015 | GO:0055085;GO:0022857;GO:0016020 | GABA permease |
| 1:5104401 | 5.08 | FPRO_01671 | intron | C | A/G | 0.090/0.090 | GO:0006355;GO:0000981;GO:0008270 | unknown protein |
| 10:2416760 | 4.77 | FPRO_13725 | extron | G | T/A | 0.045/0.015 | GO:0000160;GO:0007165;GO:0016310;GO:0055085;GO:0000155;GO:0022857;GO:0016772;GO:0016021 | hypothetical protein |
| 6:3957449 | 4.72 | – | intergenic | A | G/T | 0.060/0.015 | – |  |
| 10:2185626 | 4.67 | FPRO_13825 | intron | T | C/A | 0.090/0.030 | GO:0000160;GO:0007165;GO:0016310;GO:0055085;GO:0000155;GO:0022857;GO:0016772;GO:0016021 | hypothetical protein |
| 8:1551092 | 4.43 | FPRO_11929 | extron | C | G/A | 0.358/0.194 | GO:0004100;GO:0016758 | chitin synthase D |
| 5:763640 | 4.20 | FPRO_08500 | extron | A | G/T | 0.373/0.164 | NONE | ISWI protein |
| 2:557316 | 4.15 | – | intergenic | G | C/A | 0.030/0.015 | – |  |
| 4:649480 | 4.13 | FPRO_05744 | extron | G | C/T | 0.343/0.119 | NONE | hypothetical protein |
| 9:2488184 | 4.11 | – | intergenic | T | C/A | 0.552/0.179 | – |  |
| 2:3625209 | 4.00 | – | intergenic | A | T/C | 0.150/0.090 | – |  |
| 3:3181193 | 4.00 | FPRO_02733 | extron | G | T/C | 0.075/0.119 | NONE | DUF domain protein |
| 2:2827389 | 3.88 | FPRO_04811 | extron | G | C/T | 0.150/0.075 | NONE | hypothetical protein |
| 6:2347812 | 3.63 | FPRO_09392 | extron | T | C/G | 0.075/0.150 | GO:0006637;GO:0047617 | acyl-CoA thiolesterase |
| 11:1723531 | 3.54 | – | intergenic | G | C/T | 0.150/0.045 | – |  |
| 7:1874477 | 3.45 | – | intergenic | T | A/G | 0.075/0.150 | – |  |
| 3:1088369 | 3.45 | FPRO_03401 | extron | C | A/T | 0.150/0.090 | GO:0016020 | stomatin family protein |
| 4:3363019 | 3.42 | FPRO_06718 | extron | C | A/G | 0.150/0.090 | GO:0004497;GO:0020037;GO:0016705;GO:0005506 | cytochrome P450 |
| 4:4142359 | 3.42 | – | intergenic | G | C/A | 0.150/0.060 | – |  |
| 8:2978601 | 3.33 | – | intergenic | G | C/A | 0.045/0.075 | – |  |
| 6:2821397 | 3.33 | FPRO_09243 | extron | T | C/G | 0.060/0.134 | NONE | hypothetical protein |
| 8:845338 | 3.28 | FPRO_11708 | extron | G | T | 0.015 | NONE | hypothetical protein |
| 8:2772117 | 3.28 | FPRO_12438 | extron | C | A | 0.015 | GO:0050660;GO:0071949;GO:0016491 | 6-hydroxy-d-nicotine oxidase |
| 4:1166400 | 3.24 | – | intergenic | G | C/T | 0.150/0.090 | – |  |
| 7:1643803 | 3.24 | FPRO_10793 | extron | T | C/A | 0.090/0.150 | NONE | nucleolar complex-associated protein |
| 10:1657227 | 3.18 | FPRO_14041 | extron | A | C/T | 0.090/0.150 | GO:0005975;GO:0055085;GO:0004553;GO:0022857 | beta-glucosidase |
| 8:2182492 | 3.16 | FPRO_12199 | extron | T | G/A | 0.254/0.150 | GO:0007165 | unknown protein |
| 3:2552226 | 3.15 | – | intergenic | G | T/C | 0.090/0.045 | – |  |
| 3:4624494 | 3.12 | – | intergenic | T | A/G | 0.150/0.060 | – |  |
| 11:467792 | 3.07 | FPRO_15654 | extron | G | C/T | 0.060/0.150 | NONE | unknown protein |
| 2:586197 | 3.05 | – | intergenic | G | A/T | 0.150/0.030 | – |  |
| 1:2935488 | 3.03 | – | intergenic | A | T/C | 0.090/0.045 | – |  |
| 4:633741 | 2.98 | FPRO_05736 | extron | A | T/C | 0.150/0.104 | GO:0003824 | enoyl-CoA hydratase |
| 4:3219957 | 2.88 | FPRO_06653 | extron | G | A/T | 0.119/0.015 | GO:0005515 | MFS transporter |
| 10:1494946 | 2.86 | – | intergenic | A | G/C | 0.150/0.030 | – |  |
| 1:639420 | 2.85 | FPRO_00253 | extron | A | C/T | 0.030/0.015 | GO:0055085;GO:0022857;GO:0016020;GO:0016021 | unknown protein |
| 5:1429490 | 2.84 | – | intergenic | T | A/G | 0.150/0.075 | – |  |
| 2:3915482 | 2.77 | FPRO_05147 | extron | G | A/T | 0.119/0.045 | GO:0020037;GO:0005506;GO:0016705;GO:0004497;GO:0051536;GO:0051537;GO:0016491 | cytochrome P450 |
| 3:962262 | 2.76 | FPRO_03446 | extron | C | T/G | 0.164/0.060 | GO:0055085;GO:0005524;GO:0140359;GO:0042626;GO:0016020;GO:0016021 | ABC1 transport protein |
| 10:1076521 | 2.70 | FPRO_14276 | intron | C | G/T | 0.150/0.030 | GO:0005975;GO:0003824 | Transaldolase B |
| 1:5819127 | 2.69 | – | intergenic | C | A/T | 0.075/0.150 | – |  |
| 3:3700605 | 2.68 | FPRO_02557 | extron | A | C/T | 0.134/0.060 | GO:0003723;GO:0003676 | KRR1 protein |
| 4:3403391 | 2.65 | FPRO_06737 | extron | A | C/G | 0.150/0.075 | NONE | beta transducin-like protein |
| 2:2038189 | 2.65 | – | intergenic | G | T/C | 0.134/0.015 | – |  |
| 3:224842 | 2.64 | FPRO_03748 | extron | T | A | 0.015 | GO:0016787 | uncharacterized protein |
| 3:1224861 | 2.56 | – | intergenic | T | A/G | 0.045/0.015 | – |  |
| 2:35602 | 2.52 | FPRO_03872 | extron | C | T/G | 0.075/0.164 | GO:0008652;GO:0004072 | aspartate kinase |
| 7:1960845 | 2.46 | – | intergenic | T | G/A | 0.060/0.134 | – |  |
| 1:491549 | 2.45 | FPRO_00198 | extron | A | C/T | 0.090/0.150 | GO:0071985;GO:0005515 | palA protein |
| 1:4971000 | 2.45 | – | intergenic | T | A | 0.015 | – |  |
| 9:1619685 | 2.45 | FPRO_13084 | extron | G | T | 0.015 | GO:0006351;GO:0003677;GO:0003899;GO:0008270 | DNA-directed RNA polymerase I |
| 4:1336420 | 2.40 | FPRO_05987 | extron | C | G/T | 0.045/0.134 | GO:0005515 | anaphase control protein |
| 4:4196263 | 2.38 | FPRO_07070 | extron | A | C/T | 0.134/0.045 | NONE | unknown protein |
| 3:272594 | 2.37 | FPRO_03733 | extron | A | T/G | 0.060/0.150 | NONE | unknown protein |
| 7:336907 | 2.24 | – | intergenic | C | T | 0.015 | – |  |
| 5:4238149 | 2.24 | FPRO_07299 | extron | T | A/C | 0.045/0.015 | NONE | unknown protein |
| 3:588425 | 2.20 | – | intergenic | C | A/G | 0.164/0.075 | – |  |
| 10:342064 | 2.19 | FPRO_14588 | extron | T | G/C | 0.119/0.015 | NONE | unknown protein |
| 8:452326 | 2.17 | FPRO_11558 | extron | C | A/G | 0.150/0.104 | NONE | unknown protein |
| 10:2408739 | 2.17 | FPRO_13727 | intron | T | A/G | 0.030/0.150 | GO:0016787 | sterigmatocystin biosynthesis lipase |
| 10:2357504 | 2.15 | FPRO_13752 | intron | A | T/G | 0.015/0.015 | GO:0009308;GO:0008131;GO:0005507;GO:0048038 | copper amine oxidase 1 |
| 4:3635057 | 2.15 | FPRO_06837 | extron | A | T | 0.090 | GO:0009820;GO:0016765 | aromatic prenyltransferase |
| 1:2052381 | 2.14 | FPRO_16069 | extron | T | G/C | 0.075/0.150 | GO:0004540 | heterokaryon incompatibility protein |
| 1:6253538 | 2.13 | – | intergenic | T | A/G | 0.060/0.150 | – |  |
| 11:509901 | 2.12 | FPRO_15639 | extron | T | A/C | 0.150/0.075 | GO:0005515 | unknown protein |
| 10:2080645 | 2.10 | FPRO_13868 | extron | T | A/C | 0.030/0.134 | NONE | unknown protein |
| 3:429136 | 2.06 | FPRO_03671 | extron | A | G/T | 0.060/0.150 | GO:0005975;GO:0004553 | beta-glucosidase |
| 2:4676802 | 2.05 | FPRO_05435 | extron | C | A/T | 0.060/0.150 | GO:0003824 | amidase family protein |
| 4:3060438 | 2.04 | FPRO_06592 | extron | C | A/G | 0.134/0.045 | GO:0009308;GO:0008131;GO:0005507;GO:0048038 | peroxisomal amine oxidase |
| 6:1695154 | 2.03 | FPRO_09597 | extron | A | C/G | 0.060/0.150 | GO:0001678;GO:0005975;GO:0005536;GO:0005524;GO:0004396;GO:0016773 | hexokinase |
| 9:2462109 | 2.02 | FPRO_12810 | extron | G | T/C | 0.164/0.030 | NONE | unknown protein |
| 8:813034 | 1.99 | – | intergenic | G | A/C | 0.075/0.075 | – |  |
| 4:160563 | 1.98 | FPRO_05535 | intron | C | A/G | 0.134/0.030 | NONE | negative acting factor |
| 9:648697 | 1.96 | FPRO_13415 | extron | T | C | 0.030 | NONE | Tol protein |
| 11:1970483 | 1.93 | FPRO_15029 | extron | G | A/C | 0.134/0.060 | GO:0006629;GO:0035556;GO:0007165;GO:0008081;GO:0004435 | phospholipase C |
| 5:4285192 | 1.87 | – | intergenic | G | A/T | 0.015/0.015 | – |  |
| 8:124289 | 1.86 | – | intergenic | C | A/T | 0.045/0.045 | – |  |
| 8:2077592 | 1.85 | – | intergenic | C | T/G | 0.150/0.060 | – |  |
| 2:1718970 | 1.85 | – | intergenic | A | T/G | 0.030/0.030 | – |  |
| 8:2531698 | 1.84 | FPRO_12332 | extron | A | C/T | 0.045/0.150 | GO:0016831;GO:0016787 | 5-carboxyvanillate decarboxylase |
| 6:269204 | 1.83 | FPRO_10088 | extron | G | T/C | 0.075/0.015 | GO:0016491;GO:0008270 | ADH3-alcohol dehydrogenase III |
| 1:733158 | 1.81 | FPRO_00278 | intron | A | G/T | 0.090/0.150 | GO:0005975;GO:0004553 | probable cellulase precursor |
| 4:3226247 | 1.79 | FPRO_06657 | extron | G | A/T | 0.045/0.015 | NONE | unknown protein |
| 6:241806 | 1.72 | FPRO_10103 | extron | C | G/T | 0.015/0.015 | GO:0008194 | unknown protein |
| 10:496968 | 1.71 | – | intergenic | G | A/C | 0.150/0.015 | – |  |
| 6:3311464 | 1.70 | FPRO_09084 | extron | C | G/T | 0.164/0.045 | GO:0003824 | unknown protein |
| 7:2664949 | 1.69 | – | intergenic | A | G/C | 0.075/0.015 | – |  |
| 4:423217 | 1.67 | FPRO_05649 | extron | G | T/C | 0.150/0.015 | GO:0004497;GO:0005506;GO:0016705;GO:0020037 | cytochrome P450 |
| 1:3889668 | 1.67 | FPRO_01288 | extron | C | T/A | 0.150/0.015 | GO:0005515;GO:0003676 | argonaute like post-transcriptional gene silencing protein QDE-2 |
| 11:1357190 | 1.65 | FPRO_15276 | intron | C | A/G | 0.030/0.015 | GO:0055085;GO:0022857;GO:0016020;GO:0016021 | hexose transporter protein |
| 10:342553 | 1.64 | FPRO_14588 | extron | C | T/G | 0.150/0.045 | GO:0006355;GO:0055085;GO:0000981;GO:0008270;GO:0022857 | unknown protein |
| 4:3683523 | 1.63 | FPRO_06858 | extron | T | G/A | 0.075/0.150 | GO:0004601 | oxidase |
| 10:2149325 | 1.61 | FPRO_13840 | extron | G | A/C | 0.045/0.164 | GO:0016021 | neutral amino acid permease |
| 1:3232935 | 1.60 | – | intergenic | A | G/T | 0.134/0.075 | – |  |
| 11:461214 | 1.60 | FPRO_15656 | extron | T | A/C | 0.150/0.015 | NONE | unknown protein |
| 10:1700749 | 1.59 | FPRO_14026 | extron | T | A | 0.015 | GO:0006629 | unknown protein |
| 10:646175 | 1.59 | FPRO_14460 | extron | C | A/T | 0.104/0.179 | NONE | unknown protein |
| 11:732476 | 1.57 | FPRO_15539 | extron | C | A/G | 0.104/0.030 | GO:0055085;GO:0022857;GO:0003676 | monocarboxylate transporter 2 |
| 2:1241211 | 1.57 | FPRO_04322 | intron | C | T/A | 0.119/0.060 | NONE | ABC transport protein |
| 3:649210 | 1.56 | FPRO_03575 | extron | C | T/A | 0.045/0.015 | GO:0005515 | unknown protein |
| 3:471514 | 1.56 | FPRO_03654 | extron | G | T | 0.015 | GO:0016491 | monophenol monooxygenase |
| 10:611634 | 1.55 | FPRO_14473 | extron | C | T/G | 0.164/0.015 | GO:0006072;GO:0016491;GO:0004368;GO:0009331 | glycerol-3-phosphate dehydrogenase |
| 3:3808063 | 1.54 | FPRO_02523 | intron | G | A/C | 0.060/0.015 | NONE | actin-related protein |
| 4:2681030 | 1.52 | FPRO_06433 | intron | A | C/G | 0.164/0.045 | GO:0004064;GO:0016491 | unknown protein |
| 6:4028788 | 1.52 | FPRO_08807 | extron | C | G | 0.015 | GO:0003824 | AMP-dependent synthetase and ligase |
| 2:1744208 | 1.51 | – | intergenic | G | T/A | 0.075/0.015 | – |  |
| 2:2849681 | 1.49 | FPRO_04819 | intron | T | G/C | 0.119/0.045 | GO:0005978;GO:0004373 | glycogen synthase |
| 11:1156503 | 1.45 | – | intergenic | C | A/G | 0.164/0.045 | – |  |
| 11:1590960 | 1.44 | FPRO_15192 | extron | G | T/A | 0.150/0.060 | GO:0005975;GO:0004553 | endo-1,3-beta-glucanase |
| 11:1246721 | 1.40 | FPRO_15322 | extron | C | G/T | 0.030/0.015 | GO:0006031;GO:0004100;GO:0016758 | chitin synthase class III |
| 11:103496 | 1.38 | FPRO_15785 | extron | C | A/T | 0.164/0.030 | NONE | unknown protein |
| 6:361302 | 1.36 | FPRO_10046 | extron | A | G/C | 0.015/0.015 | GO:0043546;GO:0016491 | periplasmic nitrate reductase |
| 2:2898426 | 1.35 | – | intergenic | C | A/G | 0.150/0.045 | – |  |
| 10:1911185 | 1.35 | FPRO_13944 | extron | C | G/T | 0.045/0.150 | GO:0055085;GO:0035673 | peptide transporter |
| 12:327448 | 1.34 | FPRO_15902 | extron | T | A/G | 0.164/0.045 | GO:0009116;GO:0003824;GO:0005515 | hypothetical protein |
| 6:4031394 | 1.33 | FPRO_08807 | extron | T | G/C | 0.030/0.015 | GO:0003824 | AMP-dependent synthetase and ligase |
| 1:3582224 | 1.33 | – | intergenic | T | A/G | 0.060/0.015 | – |  |
| 9:395496 | 1.31 | FPRO_13515 | extron | A | T/C | 0.060/0.120 | GO:0003824 | unknown protein |
| 3:735406 | 1.30 | – | intergenic | C | A/G | 0.075/0.015 | – |  |
